# Innovative house structures for malaria vector control in Nampula district, Mozambique: assessing mosquito entry prevention, indoor comfort, and community acceptance

**DOI:** 10.3389/fpubh.2024.1404493

**Published:** 2024-06-04

**Authors:** Micanaldo Ernesto Francisco, Kozo Watanabe

**Affiliations:** ^1^Center for Marine Environmental Studies (CMES), Ehime University, Matsuyama, Japan; ^2^Graduate School of Science and Engineering, Ehime University, Matsuyama, Japan; ^3^Faculty of Architecture and Physical Planning (FAPF), Lurio University, Nampula, Mozambique

**Keywords:** house modification, *Anopheles*, malaria, mosquito blocking, indoor comfort

## Abstract

**Background:**

Insecticide-treated mosquito bed nets and indoor residual spraying are widely used for malaria vector control. However, their effectiveness can be affected by household members’ habits, requiring alternative approaches toward malaria vector control.

**Objective:**

To assess the effectiveness of modified houses in preventing mosquito entry; to assess the impact of house modifications on indoor air conditions and evaluate the acceptability of modified houses in the community where the study was conducted.

**Methods:**

Five traditional and five modified houses were constructed in Nampula district, Mozambique and underwent a 90-day overnight indoor mosquito collection using Centers for Disease Control and nitride ultraviolet light traps during the rainy season. Mosquitoes were identified morphologically. Indoor temperature, relative humidity, carbon dioxide levels and wind speed were also collected. The Student’s *t*-test was used to compare the means of the number of mosquitos and environmental factors between both house types. A binomial form of the Generalized Linear Model identified the factors associated with the community volunteer’s preference for house type.

**Results:**

Modified houses reduced the number of *Anopheles* by an average of 14.97 mosquitos (95% CI, 11.38–18.56, *p* < 0.000) and non-*Anopheles* by 16.66 mosquitoes (95% CI, 8.23–25.09, *p* < 0.000). Although fewer mosquitoes were trapped in modified houses compared to traditional ones, the modifications were more effective against *Anopheles* (94% reduction) than for non-*Anopheles* (71% reduction). The average temperature increased at 0.25°C in modified houses but was not statistically significant (95% CI, −0.62 to 0.12, *p* = 0.181). Community volunteers preferred modified houses due to reduced mosquito buzzing. The efficacy of modified houses including its acceptability by community, highlight its potential to lower malaria risk. Effective integration of modified houses into the vector control strategy will require raising awareness among communities about malaria risks associated with house structure and training them to modify their houses.

## 1 Introduction

*Anopheles* mosquitoes, vectors of malaria, primarily bite indoors at night (1, 2). These mosquitoes are attracted by human skin odors and carbon dioxide (CO_2_); therefore, indoor residual spraying and insecticide-treated bed nets are conventional vector control measures. Insecticides sprayed form a thin coat capable of eliminating mosquitoes resting on treated surfaces. On the other hand, mosquitoes may perish upon contact with insecticide-treated bed nets, lured by human odors toward individuals sheltered beneath them. However, the efficacy of insecticide-treated bed nets relies on consistent and correct usage as well as proper maintenance and repair (3, 4). Additionally, mosquito resistance to insecticides, acquired over time, can undermine the efficacy of both indoor residual spraying and mosquito bed nets treated with insecticides (5). Furthermore, constraints in low-income countries may limit spatial coverage of interventions. Collectively, these issues impact the overall efficacy of insecticide-treated bed nets and indoor residual spraying as reliable vector control methods (6).

Over the past decade, modifying housing structures to deter mosquito entry has garnered attention as an alternative approach to control malaria vectors. Modified houses offer pre-emptive protection against malaria by preventing mosquito infiltration indoors. *Anopheles* mosquitoes can enter through eaves, windows and doors; therefore, various blocking methods, such as installing eave tubes, blocking eaves, screening ceilings and windows and sealing gaps in doors, have been explored.

Eave tubes are sophisticated devices designed to block physically or kill mosquitoes using its electrostatic netting with powdered insecticides (7, 8). However, eave tubes need industrial manufacturing, skilled labor for installation and maintenance. These factors can limit the integration of this technology in vector control strategies, particularly in low-income and highest at-risk groups. Given that majority of families in malaria-endemic regions reside in substandard housing (9), any proposed house modification measures should be sustainable within the socio-economic constraints of these communities. Therefore, it’s essential to consider solutions that are both effective against malaria vectors and feasible within community’s socio-economic means.

Other studies using ceiling nets to block mosquitos in Gambia (10) and Kenya (11) have shown promising results in blocking mosquitoes while ensuring good ventilation inside the houses. However, ceiling nets should be provided with exact size of the compartment and properly installed by experienced labor (12). On the other hand, the nets are made of fragile material which requires proper installation and maintenance. In some regions, such as northern Mozambique, the long-term physical integrity of the nets may be compromised, given that the type of houses in this region requires regular maintenance of the roof by replacing the grass layer.

Blocking eaves (13), screening windows and doors reduces mosquito abundance indoors but affects airflow, increasing internal temperatures and reducing residents’ comfort (14). Although the gains from these modifications reduce the risk of malaria, the reduction in comfort could threaten the acceptance of these modifications by the communities as a vector control measure. Adding windows could enhance ventilation and comfort, as demonstrated in Gambia (15). However, this experiment required the installation of two screened windows and two doors on opposite walls in a single-compartment house which is not practical for Mozambican rural housing, with houses comprising more than one compartment and each compartment having only one door. The impact of house orientation toward the wind direction is worth investigating as it may potentially affect indoor ventilation and temperature. Improving indoor comfort in modified houses could influence the community to accept house modifications (16). Comfort assessments often focus solely on instrument-based measurements of environmental parameters (e.g., temperature, relative humidity and CO_2_); however, factors such as sweating intensity (17), breathing quality and perception of mosquito buzzing (18) may impact sleep quality. Hence, incorporating community volunteer interviews alongside environmental data is crucial for comprehensively assessing comfort in modified houses.

In rural communities, frequent movement in and out of houses, with doors often left open is common. Such practice may allow opportunistic mosquito entry before sleeping time, despite blocking efforts (19). To the best of our knowledge, none of previous literature investigated mechanisms to remove human interference on door closure. Thus, it is essential to integrate door-closure methods into rural house modifications to account for human behavior. Additionally, most of the previous studies that investigated the modification of houses to block mosquitoes tested only one house for each type of modification, which can reduce the robustness and confidence of the results. Evaluating the effectiveness of house modifications with replicate houses may allow for more robust data and analysis.

While house modifications typically lead to a decrease in mosquito populations, the effectiveness of these measures as a vector control strategy hinges on community willingness to adopt and implement such modifications. However, research on community acceptance of home modifications remains limited. Recognizing that communities have autonomy over their home construction, investigating their receptiveness to our proposed modifications is a critical aspect deserving attention in this study. Furthermore, engaging communities in building experimental houses can enhance project acceptability and sustainability of large scale interventions. Involving residents ensures technical knowledge transfer and autonomous implementation of house modifications. This collaborative approach allows researchers to assess residents’ learning abilities, identify strengths and weaknesses, and address potential challenges, thereby facilitating refinement and optimization of intervention strategies for sustained mosquito prevention.

This study aimed to evaluate the effectiveness of modified houses in Mozambique in preventing mosquito entry. Additionally, indoor air conditions were assessed following modifications, and community acceptance of modified houses was gauged based on residents’ sentiments regarding comfort levels.

## 2 Materials and methods

### 2.1 Study site

The experimental houses were constructed in the Murrapaniua neighborhood within Nampula district of northern Mozambique, at latitude −15.0482224^o^ and longitude 39.1918356^o^. The study site lays in a lowland, located in a rural area marked by sprawling residential growth with substandard housing and infrastructure. Nampula is one of districts with the highest incidence of malaria in Mozambique annually (20). While seasonal precipitation peaks from November to April, the peak in malaria cases occurs between January and March. Malaria is predominantly caused by *plasmodium falciparum* (21) where *An*. *funestus* s.s. and *An*. *gambiae* s.s. are the main vectors (22). The main vector control measures include indoor residual spraying programs and distribution of insecticide-treated bed nets especially to pregnant women and children (23).

**Figure 1:**
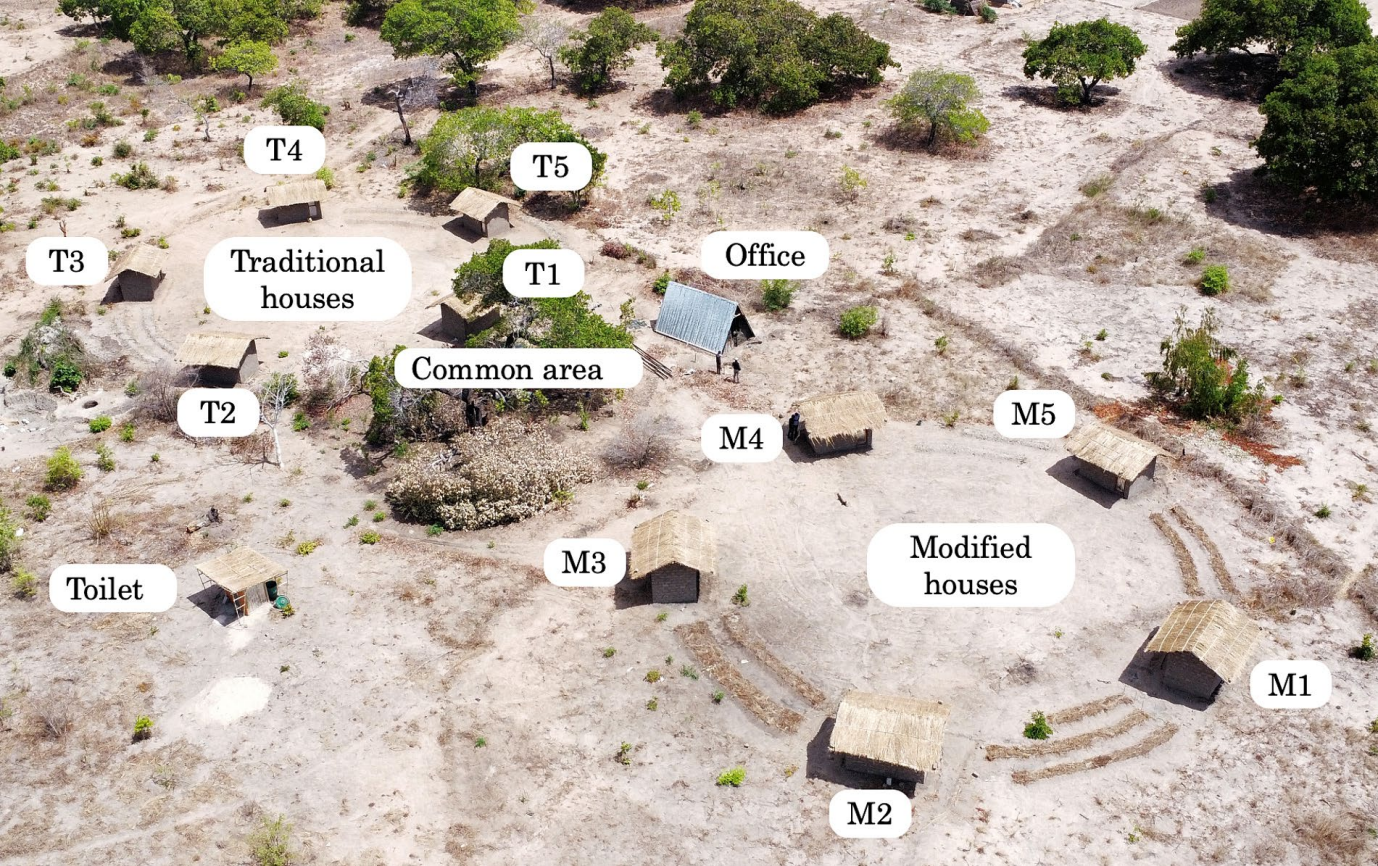
Aerial view of the experimental houses.

As shown in Figure 1, 10 experimental houses were constructed (5 traditional houses and 5 modified houses), alongside an office serving as a microscope laboratory, a communal area and a toilet. Traditional and modified houses were grouped separately, each arranged in clusters. Because traditional houses had open eaves, we expected higher release of CO_2_ outdoors compared to modified houses. Therefore, we opted placing both groups separately to avoiding potential CO_2_ from traditional houses from influencing mosquito attraction in modified houses. To ensure varied exposure to prevailing wind directions, two independent circles with a radius of 15 m were drawn and within it a pentagon polygon was inscribed. Each house was placed at a vertex of the pentagon and its front facade was oriented toward the pentagon’s center. Traditional houses were designated T1–T5, whereas modified houses were designated M1–M5. House placement in each group followed rigorous measurements to ensure the positional alignment of traditional houses and modified houses within their respective pentagons. This was to ensure each pair of houses from both traditional and modified houses was exposed to a comparable wind direction.

### 2.2 Characteristics of experimental houses

**Figure 2:**
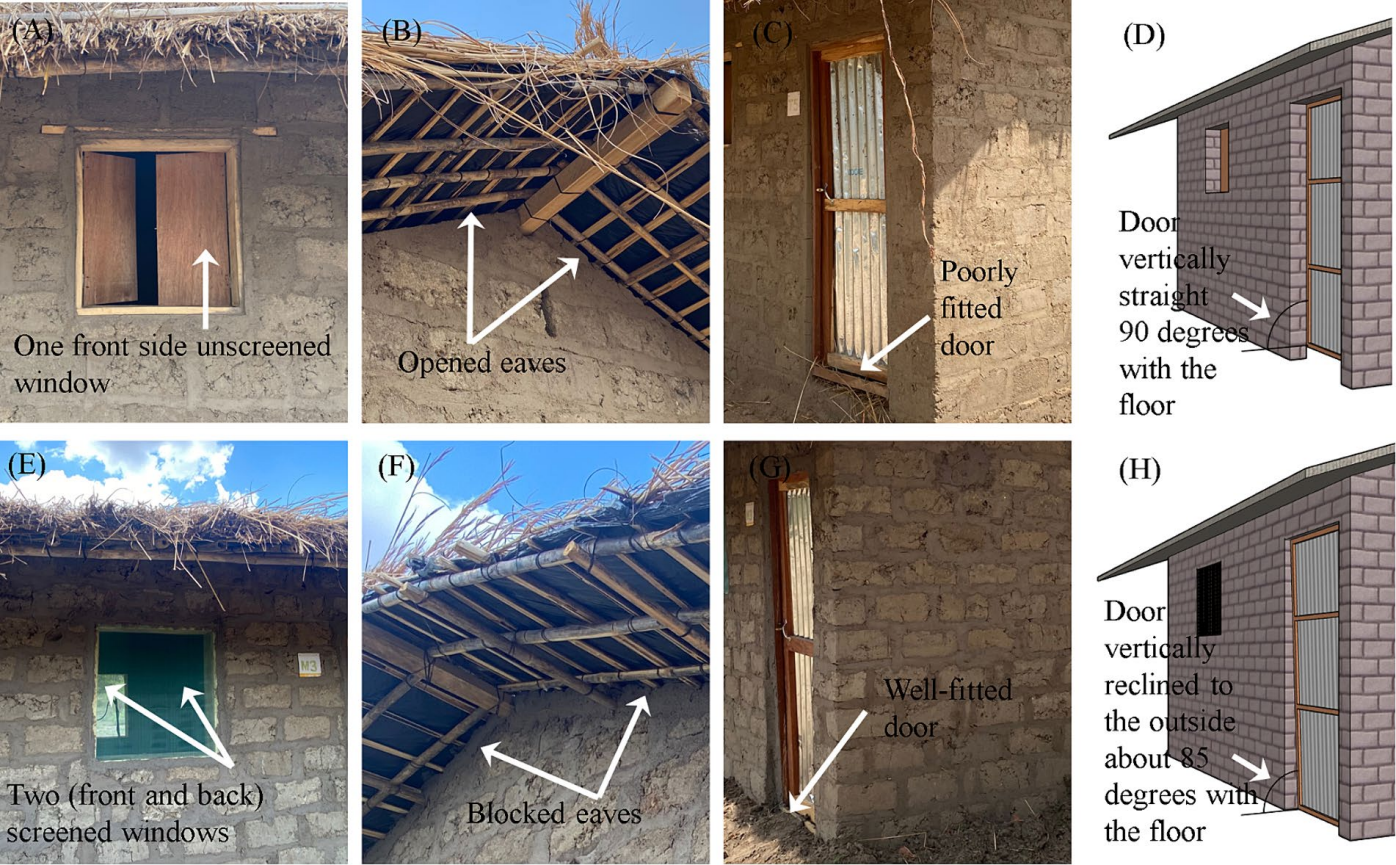
Differences between traditional and modified houses. Traditional houses featured **(A)** an unscreened window, **(B)** open eaves for ventilation, **(C)** a gap at the bottom of the door and **(D)** a vertical door position at 90° relative to the floor. Modified houses featured **(E)** screened windows, **(F)** eaves blocked with mud mortar, **(G)** no gaps at the bottom of the door and **(H)** a door slightly reclined to around 85° relative to the floor, ensuring automatic closure upon release due to a higher center of gravity.

A preliminary survey conducted in 2021 across 50 community houses facilitated the identification of the typical house design adopted for the present study. House dimensions, sleeping compartment characteristics, openings and common defects on walls and openings were recorded. For construction purposes, we adopted house size considering the average dimensions of sleeping compartments (Supplementary Table 1). Herewith, our experimental house measured 350 × 240 × 250 cm in length, width and height, respectively (Supplementary Tables 2A,B). The traditional house, representative of the standard house in the target community based on the 2021 survey, featured open eaves, a poorly fitted door measuring 70 cm in width and 180 cm in height, and a single unscreened window measuring 60 × 60 cm on the front facade (Figure 2). The modified house was created by closing the eaves with mud, adding a second window measuring 60 × 60 cm on the rear facade, screening all windows and modifying the door’s center of gravity to recline it slightly from the outside. This adjustment enabled the door to close automatically once opened and released (Supplementary Vidoe 1).

### 2.3 Construction of experimental houses

The walls of houses were built using mud blocks measuring 15 × 30 × 15 cm in width, length and height, respectively, laid with mud mortar sourced from termite mounds for enhanced resistance against heavy rain and soil moisture. Roofing consisted of slender bamboo with an average diameter of 5 cm, supported by 8 × 8 cm wooden beams resting on side walls. Bamboo layers were aligned perpendicular and parallel to the central wooden beam and spaced approximately 20 cm apart and tied together using ropes extracted from discarded tires. Dry grass was used as the roof’s finishing layer. Windows frames were made with wood and covered with plywood plate. In the modified houses the window was supplemented with mosquito net. Doors for both house types were made with wooden frames covered by corrugated metal sheets. The angle of door recline in modified houses varied as required to ensure automatic closure upon opening and release. Local artisanal construction workers, following researchers’ instructions, executed the construction. Material costs for one traditional and one modified house amounted to 104.84 and 120.47 USD, respectively. Thus, the modified house was 15% more expensive due to the addition of a second window and the screening of both windows. Additional explanation of technical terms used in this section can be found in Supplementary Table 3.

### 2.4 Questionnaire

Two separate surveys were conducted, one with 10 construction workers and another with 13 community volunteers. After completing construction of the experimental houses, construction workers were surveyed regarding their experiences applying modifications. Specifically, they were asked about the ease or difficulty of implementing modifications. The questionnaire for house community volunteer aimed to evaluate the acceptability of house modifications based on their indoor comfort experiences. Community volunteer were rotated nightly to different houses, with interviews conducted each morning. Questions covered impressions of temperature, sweating, breathing and noise intensity. Volunteers then ranked the house on a scale from 1 to 5, with higher numbers indicating greater satisfaction. Selection criteria for community volunteer included (i) residency near the experiment site in Murrapaniua neighborhood, (ii) aged 18–65 years and (iii) agreeing to sign an informed consent form. Exclusion criteria included (i) pregnant women, (ii) testing positive for COVID-19 or in close contact with infected individuals within in the past 2 weeks, (iii) individuals under medical care requiring spatial care, (iv) those unable to walk from home to the experimental site and (v) those presenting a debilitated physical condition or poor health.

### 2.5 Ethics approval

This study was approved by the Lúrio University Institutional Committee for Bioethics, Lúrio University, Mozambique, on December 9, 2021 (reference 51/Dez/CBISUL/21). The use of mosquitoes did not require ethical approval by the Ethics institution.

### 2.6 Mosquito collection and identification

This research involved human participation, with volunteers residing and sleeping in each house from 18:00 to 06:00 under informed consent. Each house was equipped with a wooden bed frame, mattress and untreated mosquito bed net. We refrained from utilizing insecticide-treated mosquito bed nests as they would kill mosquitoes and interfere on our ability to capture mosquitos with traps. Volunteers were rotated randomly among the 10 houses daily.

Two types of mosquito traps were used: five units of Centres for Disease Control (CDC) light traps (CDC Miniature Light Trap, Model 512; John W Hock Company), hereafter designated as “CDC-LTs,” and five units of nitride ultraviolet (UV) light traps, hereafter designated as “NTR-UV-LTs.” CDC-LTs are widely used for trapping mosquitoes indoors using incandescent light bulbs, whereas NTR-UV-LTs attract mosquitoes by emitting a UV light at a wavelength of 365 nm.

Traps were placed indoors, 150 cm above the ground (Supplementary Tables 2C,D), and activated at 18:00 before collection at 06:00 the next day. Trap types were alternated between house groups every 3–6 days to ensure equal exposure between traditional and modified houses. However, technical issues or atmospheric disturbances (e.g., rain) occasionally necessitated early or delayed trap interchanges. Power for NTR-UV-LTs was centralized from the office where batteries and solar panels were set, with current supplied to each house via wiring. Occasionally, we observed damaged wiring in a modified or traditional house where NTR-UV-LTs should have been installed. If repairs could not be completed before 18:00, the NTR-UV-LTs remained in the modified or traditional houses. Trap transfer to traditional or modified houses occurred the next day once technical issues were resolved. Additionally, during the initial 9 days of January, NTR-UV-LTs were not available. Consequently, we intermittently placed the CDC-LTs alone to either modified or traditional houses for a single night before transferring them to the other group the next day. This procedure persisted until January 9 when NTR-UV-LTs became available. Morphological identification of trapped anopheline mosquitoes followed the updated identification key for Afrotropical mosquitoes (24).

### 2.7 Collection of environmental data

Temperature, humidity and CO_2_ concentrations indoors were measured using the CURCONSA data logger (model MCH3). This logger operates with 5 V input power and connects to a power bank device. Data were logged every 30 min to record temperature, relative humidity and CO_2_ automatically. Measurements were stored in the built-in memory and exported to a computer via a USB cable in text (.txt) or Excel (.xlsx) formats. One data logger and an anemometer device were positioned at 150 cm above the ground and approximately 30 cm from the corner in each of the 10 houses overnight from 18:00 to 06:00 (Supplementary Tables 2C,D). Wind speed was measured using the digital anemometer (model AN-866A *CF*), powered by two AAA batteries generating 3 V in total. These devices required connection to a computer via a USB cable for real-time data recording as they lacked internal memory. One anemometer was placed at 120 cm above the ground (Supplementary Tables 2C,D) and at the center of the front window inside each of the 10 houses overnight from 18:00 to 06:00.

### 2.8 Statistical analysis

To assess the impact of house modification on indoor mosquito infestation, Student’s *t*-test analysis with two independent samples was employed to compare mean mosquito quantities in traditional versus modified houses. It is important to note that t-tests assume key assumptions for valid interpretation of results. These assumptions include normality in data distribution and equal variances (25). The *t*-test assumes that the data within each group (traditional and modified houses) follow a normal distribution. While our sample sizes were sufficiently large, we acknowledge that the mosquito count data did not perfectly meet the normality assumption based on histogram analysis. Regarding to equality of variances, we performed Levene’s test, which ensured the validity of the subsequent *t*-test results. The 95% confidence interval was used to estimate the precision of mean differences in mosquito counts between house types. Similarly, mean environmental parameter values (temperature, relative humidity, wind speed and CO_2_) between traditional and modified houses were compared using the same analytical approach. Additionally, the efficacy of modified houses in blocking *Anopheles* and non-*Anopheles* mosquitoes was compared. Efficacy in blocking *Anopheles* was calculated as follows:


$$\sum \mathrm{Anophele}s_{\mathrm{Trad}}-\sum \mathrm{Anophele}s_{\text{mod}}/\sum \mathrm{Anophele}s_{\mathrm{Trad}}\text{.}$$

Efficacy in blocking non-*Anopheles* was calculated as follows:


$$\begin{array}{l} \sum non-\mathrm{Anophele}s_{\mathrm{Trad}}-\sum non-\mathrm{Anophele}s_{\text{mod}}/\\ \sum non-\mathrm{Anophele}s_{\mathrm{Trad}}\text{,} \end{array}$$

where *Trad* and *Mod* represent traditional and modified houses, respectively.

To evaluate house modification acceptance, a rating system from 1 to 5, based on community volunteer’ feedback, was used. Ratings were further categorized into negative preference (ratings 1–2, “did not like the house”) and positive preference (rating 3–5, “liked the house”). Employing this dichotomized rank variable, the percentage of community volunteer’ responses expressing preference or disinterest in both traditional and modified houses post-sleeping experience were computed. Furthermore, a qualitative model constructed using a generalized linear model (GLM) was employed to identify factors associated with community volunteer’ preference for house type. The dichotomized rank served as the dependent variable, whereas temperature, sweating intensity, and noise intensity were employed as explanatory variables. In construction of the GLM for the qualitative model, the *family* parameter was set as “*binomial*” (26). The analysis included 595 observations. All statistical analyses were performed using R software version 4.0.2 (27).

## 3 Results

### 3.1 Comparing traditional and modified houses’ efficacy in mosquito blocking

From January 10 to March 31, CDC-LTs were used for 47 nights in traditional houses and 43 nights in modified houses. MC-UV-LTs were used for 39 nights in traditional houses and 42 nights in modified houses. There were 4 and 5 days without mosquito collection in traditional and modified houses, respectively, all occurring within the first 9 days of January (Supplementary Table 4). During the 90-day survey period (the rainy season: January 1–March 31), 21,078 female mosquitoes were collected indoors from the 10 houses. Among these, 7,535 (36%) were *Anopheles* and 13,543 (64%) were non-*Anopheles*. *Anopheles* mosquitoes were predominately collected in traditional houses (7,135) compared with modified houses (400). Similarly, traditional houses yielded a higher number of non-*Anopheles* mosquitoes (10,520) compared with modified houses (3,023; Table 1). Overall, modified houses exhibited 94% fewer *Anopheles* and 71% fewer non-*Anopheles* mosquitoes on average compared with traditional houses.

**Table 1:** Distribution of collected female mosquitoes per house.

House	*Anopheles* mosquito						Non-*Anopheles* mosquito	
	*An. gambiae s.l.*	*An. funestus s.l.*	*An. rufipes*	*An. coustani*	Sub-total	Total	Sub-total	Total
T1	963	523	9	3	1,498	7,135	2,330	10,520
T2	900	588	5	1	1,494		2,111	
T3	772	532	3	8	1,315		1,992	
T4	685	444	5	3	1,137		1,879	
T5	1,126	563	2	0	1,691		2,208	
M1	29	70	0	4	103	400	735	3,023
M2	25	47	0	2	74		437	
M3	32	53	0	0	85		673	
M4	23	42	0	1	66		544	
M5	27	44	1	0	72		634	
Total	4,582	3,346	25	22	7,535	7,535	13,543	13,543

T, traditional house; M, modified house.

**Figure 3:**
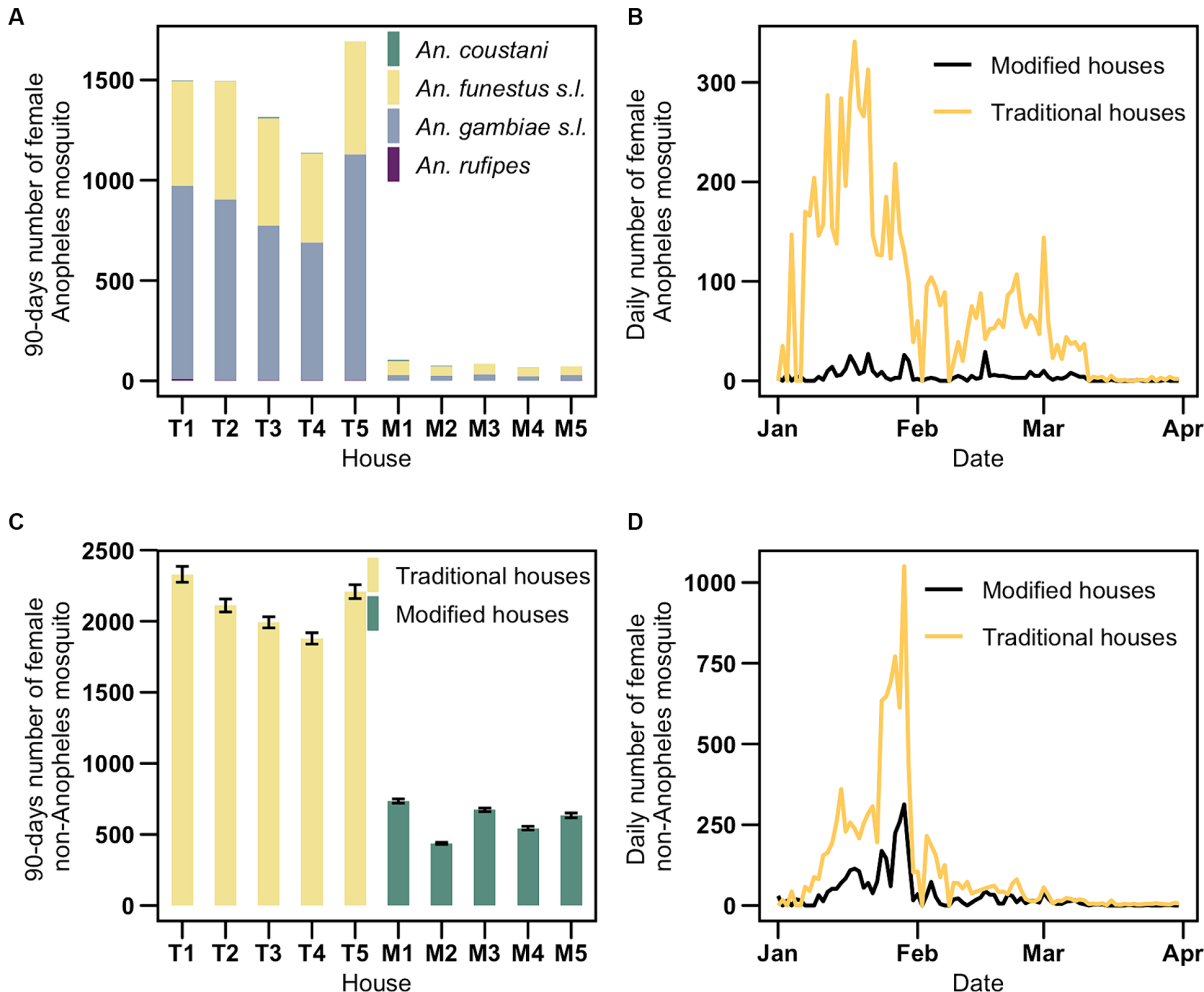
Mosquito abundance from January to March. **(A)** Female *Anopheles* mosquito distribution per traditional and modified house. **(B)** Daily distribution of female *Anopheles* mosquitoes in traditional and modified houses. **(C)** Female non-*Anopheles* mosquito distribution per traditional and modified house. **(D)** Daily distribution of female non-*Anopheles* mosquitoes in traditional and modified houses. T and M indicate traditional and modified houses, respectively.

In traditional houses, T5 and T1 were the most penetrable for *Anopheles*, yielding 1,691 and 1,498 mosquitoes, respectively. A similar trend was observed for non-*Anopheles* mosquitoes, with T1 and T5 yielding 2,330 and 2,208 non-*Anopheles* mosquitoes, respectively. Among the modified houses, M1 and M3 were the most penetrable for both *Anopheles* (103 and 85 mosquitoes, respectively) and non-*Anopheles* (735 and 673 mosquitoes, respectively; Figures 3A,B). *Anopheles* mosquitoes peaked in mid-January, whereas non-*Anopheles* mosquitoes peaked at the end of January, with both peaks following after the initial rainfall peak in January (Supplementary Image 1).

Student’s *t*-test analysis revealed significant reductions in *Anopheles* mosquitoes indoors in modified houses compared with traditional houses, with an average reduction of 14.97 mosquitoes (95% CI: 11.38–18.56; *p* < 0.001; Table 2). Similarly, non-*Anopheles* mosquitoes in modified houses compared with traditional houses, with an average reduction of 16.66 mosquitoes (95% CI: 8.23–25.09; *p* < 0.001).

**Table 2:** Mosquito density and indoor comfort compared between traditional and modified houses using Student’s *t*-test.

Variable	Average values and range		95% CI	*p*-value
	Traditional houses	Modified houses		
Female *Anopheles* mosquito	15.86 (0 to 68.2)	0.89 (0 to 5.8)	14.97 (11.38 to 18.56)	<0.000
Female non-*Anopheles* mosquito	23.38 (0 to 210)	6.72 (0 to 62.6)	16.66 (8.23 to 25.09)	<0.000
Carbon dioxide (ppm)	443.68 (389.00 to 545.74)	459.63 (385.75 to 678.03)	−15.95 (−30.43 to −1.48)	0.031
Temperature (°C)	25.09 (22.47 to 28.52)	25.34 (22.83 to 29.59)	−0.25 (−0.62 to 0.12)	0.181
Relative humidity (%)	67.14 (58.90 to 73.96)	66.14 (56.82 to 76.18)	1.00 (−0.13 to 2.14)	0.082
Wind speed (m/s)	0.15 (0.01 to 0.57)	0.22 (0 to 0.68)	−0.07 (−0.13 to −0.02)	0.006

CI, confidence interval.

### 3.2 Assessment of house modifications’ impact on indoor air conditions

CO_2_ concentrations increased by 15.95 ppm in modified houses compared with traditional houses (95% CI: −30.43 to −1.48; *p* = 0.031; Table 2). Although temperature increased by 0.25°C in modified houses, the difference between modified and traditional houses was not statistically significant (95% CI: −0.62 to 0.12; *p* = 0.181). There was 1% less moisture in modified houses than in traditional houses; however, the difference was not statistically significant (95% CI: −0.13 to 2.14; *p* = 0.082). Airflow in modified houses marginally but significantly improved by 0.07 m/s compared with traditional houses (95% CI: −0.13 to −0.02; *p* = 0.006). Comparisons between the five houses within each group regarding mosquito infestation and environmental parameters revealed non-significant differences for all variables, except wind speed in both traditional (*p* = 0.026) and modified (*p* < 0.000) houses (Supplementary Table 5).

### 3.3 Evaluation of modified house acceptability

For both traditional and modified houses, a maximum number of *n* = 450 sleeps was recorded for each group. In traditional and modified houses, there were 297 (66.0%) and 298 (66.2%) sleeps, respectively, after 90 days. Results indicated a clear preference for modified houses over traditional houses. Regarding traditional houses, community volunteers were interviewed 297 times, with interviewees on 42 occasions (14%) expressing a liking for the house, whereas 255 (86%) expressions of dislike were recorded. Regarding modified houses, community volunteers were interviewed 298 times, with 272 (91%) expressions of preference for these houses, whereas only 26 (9%) responses indicated dislike. As shown in Table 3, the GLM model (Akaike information criterion: 663.64) indicated that noise intensity (mosquito buzzing) was the most influential factor affecting a community volunteer’s house preference (*β* = −0.712, *p* < 0.001) followed by temperature (*β* = −0.431, *p* = 0.025). Sweating intensity exerted no significant impact on a community volunteer’s preference (*β* = −0.117, *p* = 0.553).

**Table 3:** Generalized linear model results regarding community volunteers’ preferences toward traditional and modified houses.

Variable	Coefficient	*p*-value
(Intercept)	2.862	<0.000
Temperature level	−0.431	0.025
Sweating intensity	−0.117	0.553
Noise intensity	−0.712	<0.000

Noise: humming sounds emitted by mosquitoes. All explanatory variables were categorical, with values of 1, 2, 3, 4, and 5 corresponding to very low, low, moderate, high and very high ratings, respectively.

### 3.4 Assessment of construction workers’ experience in building modified houses

All construction workers were recruited from nearby communities, with 90% having no formal education or training in construction (Supplementary Table 6). However, all construction workers had substantial experience in constructing traditional houses in their community. Constructing modified houses involved several new modifications, including optimizing the orientation for maximum wind exposure at the window, adding mosquito nets to windows, blocking eaves with mud, reclining doors outward and installing door stoppers. Although most modifications were unfamiliar to the construction workers, after following the researchers’ guidelines, all stated that they could apply these modifications independently. However, some challenges were encountered, with 50% of construction workers finding reclining doors and closing eaves easy and the other 50% finding these processes difficult (Supplementary Table 6). Blocking eaves with mud mortar was challenging when the bamboo roof structure was oblique relative to the eave gaps. We found that the bamboo in longitudinal direction above the eave gaps must be aligned parallel to the respective walls to facilitate filling with mud mortar. Similarly, outward door reclining was found easier to achieve with straight walls (90° relative to the ground) or slightly outward-reclining walls. However, inward-reclined walls posed challenges for fitting doors to close automatically upon release. On the other hand, inconsistent use of tools, such as spirit levels and plumb bobs, led to crooked wall construction.

## 4 Discussion

The number of mosquitoes in modified houses was significantly lower than that in traditional houses, demonstrating the efficacy of house modifications in blocking mosquito entry. Screening windows and blocking eaves provides a physical barrier preventing mosquitoes from entering indoor environments (28, 29). Additionally, blocking eaves reduces the attraction of mosquitoes, which are drawn to human odors and CO_2_ (30). By blocking eaves, the outdoor release of these attractants is minimized, making the house less appealing to mosquitoes (13). Additionally, we introduced a novel modification by reclining the door at approximately 85° outward, using the principle of the center of gravity to create an automatic door closing mechanism. This feature eliminates the need for manual door closure, ensuring consistent closure and effectively preventing opportunistic mosquito entry via an open door. This modification may offer enhanced protection for individuals in rural areas, who frequently move in and out of the house during daily activities while keeping the door opened.

House modifications may provide a supplemental, effective and sustainable approach to preventing malaria infections compared with conventional methods, such as insecticide-treated bed nets and indoor residual spraying. Although insecticide-treated bed nets require consistent and correct usage for optimal efficacy, their long-term effectiveness can be hindered by human-related factors. Household environment–related behaviors (e.g., storing goods in sleeping rooms, sleeping on the floor, using specific bed frame types and sharing of the same insecticide-treated bed net by several individuals) and net care–related behaviors (e.g., improper folding and washing maintenance) have been reported to affect the longevity and long-term efficacy of insecticide-treated bed nets (31, 32). House modifications provide more efficient protection by addressing entry points and minimizing the risk of mosquito infiltration, regardless of sleeping environment conditions, as well as reducing human interference. Additionally, indoor residual spraying faces several challenges, including the potential environmental impact, limited coverage, insecticide resistance (33, 34) and interference by household owners (e.g., renovations, such roof changes or wall plastering), decreasing the efficacy of spraying. In contrast, house modifications eliminate the reliance on insecticides, offering a safe and environmentally friendly solution that does not require intensive care and removes human interference.

Modified houses proved more effective in reducing *Anopheles* mosquitoes entry compared with non-*Anopheles* mosquitoes. Gaps in eaves and doors were blocked, and windows were properly screened, targeting all expected entry points for all mosquito genera; however, non-*Anopheles* mosquitoes still entered at a higher ratio. The mechanism underlying this difference remains unclear. A previous study suggested that this phenomenon may be explained by differences in entry point preferences between *Anopheles* (eaves) and non-*Anopheles* mosquitoes (windows and doors) (35). Based on this assumption, targeting all potential entry points (eaves, doors and windows) would theoretically eliminate the divergent entry ratio across all mosquito genera. However, our findings did not support this assumption, despite our modifications targeting all potential entry points for all mosquito genera. Our results align with a recent study reporting a higher entry ratio of non-*Anopheles* mosquitoes compared with *Anopheles* mosquitoes, even after eaves, doors and windows were properly fitted and screened (36). Although malaria transmission relies on *Anopheles* mosquitoes, non-*Anopheles* infestation may increase nuisance biting and buzzing noises. Therefore, further research is necessary to reduce non-*Anopheles* mosquito entry for improved comfort and sleeping quality. Nonetheless, our house design effectively reduces malaria vector mosquito entry, demonstrating a high potential in mitigating malaria risk in impoverished housing.

Although house modifications effectively reduced mosquito entry, indoor comfort was negatively affected. In modified houses, air temperature and CO_2_ concentrations were increased, whereas relative humidity decreased. Despite the temperature rising by 0.25°C, lower than that reported in previous studies (10, 36), this outcome contradicted our expectations, considering the addition of a new window on the rear wall to compensate for the blocked eaves. The air temperature increase and CO_2_, along with the decrease in relative humidity in modified houses may be attributed to air physics. Warmer air is less dense and tends to rise with temperature increases (37); hence, in modified houses, warmer air, along with higher concentrations of CO_2_, accumulated at the ceiling due to the blocked eaves. This accumulation likely decreased air moisture levels, lowering relative humidity. However, these changes are unlikely to exert major effects on health. Although adding a window at the rear facade improved airflow by an average of 0.07 m/s, it did not significantly affect other environmental parameters. This suggests that aligning windows on opposing walls may enhance horizontal airflow but does not lead to substantial circulation of air at the top of eaves. Open eaves can more efficiently expel warmer air compared with windows (38) while also allowing warmer indoor air to mix with cooler external air, thereby improving air conditions in traditional houses. Nevertheless, our findings indicate that despite minor changes in indoor comfort, the benefits of reduced mosquito entry in modified houses outweigh these inconveniences.

In this study, we also focused on community volunteer’s preferences, particularly emphasizing comfort differences between modified and traditional houses. Results revealed that community volunteers favored modified houses, with noise intensity (mosquito buzzing) cited as the primary influencing factor. Although previous studies have highlighted temperature as a crucial factor in determining overall satisfaction with a living environment (39, 40), our findings indicate the importance of mosquito buzzing in determining sleep quality and comfort. The temperature variation between house types was minimal and insignificant such that temperature was not a factor for evaluating indoors comfort in our study. Furthermore, the relative increase in CO_2_ and relative humidity by 15.95 ppm and 1% average respectively, was not sufficient to affect indoor comfort that could be felt by community volunteers. Therefore, we believe that none of the environmental factors played a significant role in community volunteer’s assessment of comfort, but mosquito buzzing.

The involvement of local construction workers and their ability to learn the construction process is vital for sustainability and community engagement. Local artisanal construction workers lacked formal training but drew on experience building traditional adobe houses to apply modifications, highlighting the potential for knowledge transfer within the community through these construction workers. In the process of modifying houses, we found that proper alignment of bamboo with wall in the longitudinal direction above eave gaps is crucial for effective sealing with mud mortar, as oblique bamboo complicate the process. This technique, once mastered, significantly enhances the quality of eave gap seals. Additionally, door installation was more straightforward with straight or slightly outward-reclining walls, whereas inward-reclining walls hindered automatic door closure. Overall, these insights highlight the need for proper construction techniques and tool usage to improve building quality and functionality. The involvement of residents was limited to those invited as construction workers. Although their prior experience appeared to facilitate their process of learning to apply modifications, our study design, the number of construction workers and the number of modified houses were insufficient for a solid assessment of potential weaknesses and strengths in training local residents to modify houses. Future studies should focus on developing larger scale training programs including evaluation mechanisms in aiding local communities in constructing modified houses.

The successful implementation of modified houses as a vector control strategy relies heavily on the cooperation of local communities, as they have control over house construction. Community involvement is not only critical but also essential for the long-term sustainability of the project. Unlike indoor residual spraying and mass distribution of insecticide-treated mosquito bed nets, which are ongoing expenses borne by government agencies, house modification offers the potential for sustainable vector control. To initiate the integration of modified houses as a vector control strategy, it is crucial to equip communities with knowledge about the risk factors associated with malaria infection related to house structures. It is essential to foster community understanding of the necessity of building modifications to safeguard their well-being and protect against mosquitoes. Subsequently, community training becomes vital in providing the necessary knowledge and skills for implementing house modifications effectively. Without community recognition of the importance of modified houses in mosquito control and proficiency in modification techniques, the effectiveness of utilizing modified houses for vector control will be compromised. The house modifications implemented in this study may be promising for replication in regions with similar building materials and house types. We believe that adaptations will be necessary for implementation in areas with different construction materials and housing characteristics. Therefore, adjustments and customization are vital to tailor modified house strategies to specific regional socio-economic contexts, ensuring effective vector control.

The study’s design, involving multiple modifications in all modified houses, prevented us to measure the specific impact of each modification. For example, the effectiveness of adding the automated door closure against mosquitoes could not be quantified in this study. Historically, studies have achieved notable reductions in mosquito entry, typically ranging between 94 and 96% with window screening and eaves blocking (36). Hence, we anticipated that incorporating door closures would yield even higher blocking rates than those reported in prior research. However, our findings did not demonstrate any improvement. This limitation underscores the need for future research to discerning which modification has major impact on blocking mosquito entry.

Our statistical tests revealed no significant differences when comparing mosquito numbers and environmental parameters among the five houses within traditional or modified groups, testing the potential effect of house orientation toward the prevailing wind direction. Thus, house orientation may not significantly influence mosquito infestation and environmental parameters. Consequently, future studies with comparable objectives may reasonably disregard house orientation as a significant factor, as our findings indicate that assessing only one house could capture general trends in mosquito numbers and environmental conditions for a specific house design. Despite our efforts to adhere to statistical assumptions, it is important to acknowledge potential limitations and biases that could influence the interpretation of our results. Our experimental houses were limited to the type of building common in northern Mozambique. Our results may not be fully representative of broader populations like the other regions of Mozambique where the type of buildings are different. Although we compared mean environmental parameters between house types, the inconsistent presence of community volunteer and other unmeasured environmental variables (e.g., presence of standing water, vegetation density) could have influenced mosquito infestation levels, potentially confounding our results. The cross-sectional nature of our study limits causal inference. Longitudinal studies would provide more robust evidence of the impact of house modifications on mosquito infestation over time. To address these limitations, future research could incorporate larger and more diverse study populations, control for additional environmental factors, and employ longitudinal study designs to capture temporal trends in mosquito infestation post-modification.

## 5 Conclusion

This study provides evidence of modified houses’ effectiveness in reducing mosquito entry, particularly *Anopheles* species, which are malaria vectors. Modified houses can complement insecticide-treated bed nets and indoor residual spraying for robust malaria vector control. Effective integration of modified houses into the vector control strategy will require raising awareness among communities about malaria risks associated with house structure and training them to modify their houses and encourage its adoption. Community engagement is crucial for the sustainability of house modifications for vector control at long term. However, this approach requires policy support and community interventions to encourage house modifications. Collaborations among governments, non-governmental organizations and community leaders are essential. A comprehensive approach, including modified houses, insecticide-treated bed nets and indoor residual spraying, could markedly reduce malaria transmission.

## Data availability statement

The raw data supporting the conclusions of this article will be made available by the authors, without undue reservation.

## Ethics statement

The studies involving humans were approved by Comité Institucional de Bioética para a Saúde da Universidade Lúrio, Lurio University. The studies were conducted in accordance with the local legislation and institutional requirements. The participants provided their written informed consent to participate in this study.

## Author contributions

MF: Writing – review & editing, Writing – original draft, Visualization, Software, Methodology, Investigation, Formal analysis, Data curation, Conceptualization. KW: Writing – review & editing, Validation, Supervision, Resources, Project administration, Funding acquisition, Conceptualization.

## Supplementary material

SUPPLEMENTARY IMAGE 1 Daily mean values of environmental factors. Gray solid line is traditional house and black solid line is modified house. **(A)** is daily mean temperature in degrees Celsius. **(B)** is daily mean carbon dioxide in parts per million. **(C)** is daily mean relative humidity in percentage. **(D)** is daily mean wind speed in meters per second. **(E)** is daily precipitation in millimeters per day. All environmental factors were measured indoors except for precipitation measured outdoors.

## References

[1] Cooke MK Kahindi SC Oriango RM Owaga C Ayoma E Mabuka D. 'A bite before bed': exposure to malaria vectors outside the times of net use in the highlands of western Kenya. Malar J. (2015) 14:259. doi: 10.1186/s12936-015-0766-4, PMID: 26109384 PMC4479228

[2] Mburu MM Mzilahowa T Amoah B Chifundo D Phiri KS van den Berg H. Biting patterns of malaria vectors of the lower Shire valley, southern Malawi. Acta Trop. (2019) 197:105059. doi: 10.1016/j.actatropica.2019.105059, PMID: 31194960

[3] Iwashita H Dida G Futami K Sonye G Kaneko S Horio M. Sleeping arrangement and house structure affect bed net use in villages along Lake Victoria. Malar J. (2010) 9:176. doi: 10.1186/1475-2875-9-176 20569459 PMC2906499

[4] Mosha JF Lukole E Charlwood JD Wright A Rowland M Bullock O. Risk factors for malaria infection prevalence and household vector density between mass distribution campaigns of long-lasting insecticidal nets in North-Western Tanzania. Malar J. (2020) 19:297. doi: 10.1186/s12936-020-03369-4, PMID: 32819368 PMC7441624

[5] Wanjala CL Zhou G Mbugi J Simbauni J Afrane YA Ototo E. Insecticidal decay effects of long-lasting insecticide nets and indoor residual spraying on Anopheles gambiae and Anopheles arabiensis in Western Kenya. Parasit Vectors. (2015) 8:588. doi: 10.1186/s13071-015-1194-6, PMID: 26567915 PMC4644290

[6] Chaccour CJ Alonso S Zulliger R Wagman J Saifodine A Candrinho B. Combination of indoor residual spraying with long-lasting insecticide-insecticidetreated Zambezia, Mozambique: a cluster randomised trial and cost-effectiveness study protocol. BMJ Globa Health. (2018) 3:e000610:1–8. doi: 10.1136/bmjgh-2017-000610 PMC5859815 29564161

[7] Snetselaar J Njiru BN Gachie B Owigo P Andriessen R Glunt K. Eave tubes for malaria control in Africa: prototyping and evaluation against *Anopheles gambiae* s.s. and Anopheles arabiensis under semi-field conditions in western Kenya. Malar J. (2017) 16:276. doi: 10.1186/s12936-017-1926-5, PMID: 28778169 PMC5545004

[8] Sternberg ED Ng’habi KR Lyimo IN Kessy ST Farenhorst M Thomas MB. Eave tubes for malaria control in Africa: initial development and semi-field evaluations in Tanzania. Malar J. (2016) 15:447. doi: 10.1186/s12936-016-1499-8, PMID: 27586055 PMC5009540

[9] Takarinda KP Nyadundu S Govha E Gombe NT Chadambuka A Juru T. Factors associated with a malaria outbreak at Tongogara refugee camp in Chipinge District, Zimbabwe, 2021: a case–control study. Malar J. (2022) 21:94. doi: 10.1186/s12936-022-04106-9, PMID: 35305666 PMC8933855

[10] Lindsay SW Jawara M Paine K Pinder M Walraven GEL Emerson PM. Changes in house design reduce exposure to malaria mosquitoes. Trop Med Int Health. (2003) 8:512–7. doi: 10.1046/j.1365-3156.2003.01059.x, PMID: 12791056

[11] Minakawa N Kawada H Kongere JO Sonye GO Lutiali PA Awuor B. Effectiveness of screened ceilings over the current best practice in reducing malaria prevalence in western Kenya: a cluster randomized-controlled trial. Parasitology. (2022) 149:944–55. doi: 10.1017/S0031182022000415, PMID: 35437129 PMC10090608

[12] Kagaya W Chan CW Kongere J Kanoi BN Ngara M Omondi P. Evaluation of the protective efficacy of Olyset®plus ceiling net on reducing malaria prevalence in children in Lake Victoria Basin, Kenya: study protocol for a cluster-randomized controlled tri. Trials. (2023) 24:354. doi: 10.1186/s13063-023-07372-3, PMID: 37231429 PMC10210418

[13] Mburu MM Juurlink M Spitzen J Moraga P Hiscox A Mzilahowa T. Impact of partially and fully closed eaves on house entry rates by mosquitoes. Parasit Vectors. (2018) 11:383. doi: 10.1186/s13071-018-2977-3, PMID: 29970153 PMC6029021

[14] Kua KP Lee SWH. Randomized trials of housing interventions to prevent malaria and Aedes-transmitted diseases: a systematic review and metaanalysis. PLoS One. (2021) 16:e0244284. doi: 10.1371/journal.pone.0244284, PMID: 33417600 PMC7793286

[15] Jatta E Carrasco-Tenezaca M Jawara M Bradley J Ceesay S D'Alessandro U. Impact of increased ventilation on indoor temperature and malaria mosquito density: an experimental study in the Gambia. J R Soc Interface. (2021) 18:20201030. doi: 10.1098/rsif.2020.1030, PMID: 33975463 PMC8113914

[16] Sternberg ED Cook J Ahoua Alou LP Aoura CJ Assi SB Doudou DT. Evaluating the impact of screening plus eave tubes on malaria transmission compared to current best practice in central Côte d’Ivoire: a two armed cluster randomized controlled trial. BMC Public Health. (2018) 18:894. doi: 10.1186/s12889-018-5746-5, PMID: 30021543 PMC6052618

[17] Mold JW Woolley JH Nagykaldi Z. Associations between night sweats and other sleep disturbances: an OKPRN study. Ann Fam Med. (2006) 4:423–6. doi: 10.1370/afm.554, PMID: 17003142 PMC1578640

[18] Kılıç C Öz G Avanoğlu KB Aksoy S. The prevalence and characteristics of misophonia in Ankara, Turkey: population-based study. BJPsych Open. (2021) 7:e144. doi: 10.1192/bjo.2021.978, PMID: 34353403 PMC8358974

[19] Barreaux AMG Oumbouke WA Brou N'G Tia IZ Ahoua Alou LP Doudou DT. The role of human and mosquito behaviour in the efficacy of a house-based intervention. Philos Trans R Soc Lond Ser B Biol Sci. (2021) 376:20190815. doi: 10.1098/rstb.2019.0815, PMID: 33357057 PMC7776932

[20] Harp RD Colborn JM Candrinho B Colborn KL Zhang L Karnauskas KB. Interannual climate variability and malaria in Mozambique. GeoHealth. (2021) 5:e2020GH000322. doi: 10.1029/2020GH000322

[21] Mabunda S Casimiro S Quinto L Alonso P. A country-wide malaria survey in Mozambique. I. Plasmodium falciparum infection in children in different epidemiological settings. Malar J. (2008) 7:216. doi: 10.1186/1475-2875-7-216, PMID: 18950486 PMC2579920

[22] The PMI VectorLink Project Mozambique. Mozambique entomological monitoring annual report. Rockville, MD: The PMI VectorLink Project, Abt Associates Inc (2020).

[23] PMI (n.d). Mozambique. Available at: https://pmivectorlink.org/where-we-work/mozambique/ (Accessed May 13, 2024).

[24] Coetzee M. Key to the females of Afrotropical Anopheles mosquitoes (Diptera: Culicidae). Malar J. (2020) 19:70. doi: 10.1186/s12936-020-3144-9, PMID: 32054502 PMC7020601

[25] Kim TK. T test as a parametric statistic. Korean J Anesthesiol. (2015) 68:540–6. doi: 10.4097/kjae.2015.68.6.540 26634076 PMC4667138

[26] de Souza R Cameron E Killedar M Hilbe J Vilalta R Maio U. The overlooked potential of generalized linear models in astronomy, I: binomial regression. Astron Comput. (2015) 12:21–32. doi: 10.1016/j.ascom.2015.04.002

[27] R Core Team. R: a language and environment for statistical computing. Vienna: (2022).

[28] Kaindoa EW Finda M Kiplagat J Mkandawile G Nyoni A Coetzee M. Housing gaps, mosquitoes and public viewpoints: a mixed methods assessment of relationships between house characteristics, malaria vector biting risk and community perspectives in rural Tanzania. Malar J. (2018) 17:298. doi: 10.1186/s12936-018-2450-y, PMID: 30119666 PMC6098617

[29] Ngadjeu CS Doumbe-Belisse P Talipouo A Djamouko-Djonkam L Awono-Ambene P Kekeunou S. Influence of house characteristics on mosquito distribution and malaria transmission in the city of Yaoundé, Cameroon. Malar J. (2020) 19:53. doi: 10.1186/s12936-020-3133-z, PMID: 32000786 PMC6993434

[30] Webster B Lacey E Cardé R. Waiting with bated breath: opportunistic orientation to human odor in the malaria mosquito, *Anopheles gambiae*, is modulated by minute changes in carbon dioxide concentration. J Chem Ecol. (2015) 41:59–66. doi: 10.1007/s10886-014-0542-x, PMID: 25572756

[31] Abílio AP Obi E Koenker H Babalola S Saifodine A Zulliger R. Monitoring the durability of the long-lasting insecticidal nets MAGNet and Royal Sentry in three ecological zones of Mozambique. Malar J. (2020) 19:209–17. doi: 10.1186/s12936-020-03282-w, PMID: 32552819 PMC7301518

[32] Minakawa N Kongere JO Dida GO Ikeda E Hu J Minagawa K. Sleeping on the floor decreases insecticide treated bed net use and increases risk of malaria in children under 5 years of age in Mbita District, Kenya. Parasitol. (2015) 142:1516–22. doi: 10.1017/S0031182015000955, PMID: 26282826

[33] Barbosa S Kay K Chitnis N Hastings IM. Modelling the impact of insecticide-based control interventions on the evolution of insecticide resistance and disease transmission. Parasit Vectors. BMC Parasites & Vectors. (2018) 11:482. doi: 10.1186/s13071-018-3025-z, PMID: 30153869 PMC6114906

[34] Tangena J-AA Hendriks CMJ Devine M Tammaro M Trett AE Williams I. Indoor residual spraying for malaria control in sub-Saharan Africa 1997 to 2017: an adjusted retrospective analysis. Malar J. (2020) 19:150. doi: 10.1186/s12936-020-03216-6, PMID: 32276585 PMC7149868

[35] Njie M Dilger E Lindsay SW Kirby MJ. Importance of eaves to house entry by Anopheline, but not Culicine, Mosquitoes. J Med Entomol. (2009) 46:505–10. doi: 10.1603/033.046.0314, PMID: 19496420

[36] Jatta E Jawara M Bradley J Jeffries D Kandeh B Knudsen JB. How house design affects malaria mosquito density, temperature, and relative humidity: an experimental study in rural Gambia. Lancet Planet Earth. (2018) 2:e498–508. doi: 10.1016/S2542-5196(18)30234-1, PMID: 30396441

[37] Liu J Li Z Kim MK Zhu S Zhang L Srebric J. A comparison of the thermal comfort performances of a radiation floor cooling system when combinedwith a range of ventilation systems. Indoor Built Environ. (2019) 29:527–42. doi: 10.1177/1420326X19869412

[38] Knudsen JB Pinder M Jatta E Jawara M Yousuf MA Søndergaard AT. Measuring ventilation in different typologies of rural Gambian houses: a pilot experimental study. Malar J. (2020) 19:273. doi: 10.1186/s12936-020-03327-0, PMID: 32736629 PMC7393878

[39] Carrasco-Tenezaca M Jawara M Lee DSH Holmes MS Ceesay S McCall P. Effect of passive and active ventilation on malaria mosquito house entry and human comfort: an experimental study in rural Gambia. J R Soc Interface. (2023) 20:20220794. doi: 10.1098/rsif.2022.0794, PMID: 37015266 PMC10072938

[40] Loughnan M Carroll M Tapper NJ. The relationship between housing and heat wave resilience in older people. Int J Biometeorol. (2015) 59:1291–8. doi: 10.1007/s00484-014-0939-9, PMID: 25523613

