# Supplementary material for: Innovative house structures for malaria vector control in Nampula district, Mozambique: assessing mosquito entry prevention, indoor comfort, and community acceptance
Source: Front Public Health. 2024 Jun 4;12:1404493. doi: 10.3389/fpubh.2024.1404493 (PMC11183294; doi:10.3389/fpubh.2024.1404493)
Supplement: Supplementary file 1 [file Table_1.docx]

Supplemental Table 1: Summary of results from preliminary survey in 2021.

| **Property status** | **n** | **Percentage** |
| --- | --- | --- |
| Self-built | 42 | 84% |
| Renting | 4 | 8% |
| Inherited | 4 | 8% |

| **Number of compartments** | **n** | **Percentage** |
| --- | --- | --- |
| One | 3 | 6% |
| Two | 13 | 26% |
| Three | 24 | 48% |
| Four | 9 | 18% |
| Five | 1 | 2% |

| **House width** | **in cm** |
| --- | --- |
| Minimum | 360 |
| Maximum | 1440 |
| Mean | 510.51 |
| Median | 490 |
| Standard deviation | 147.2 |

| **House length** | **in cm** |
| --- | --- |
| Minimum | 500 |
| Maximum | 1720 |
| Mean | 667.08 |
| Median | 620 |
| Standard deviation | 186.61 |

| **Ceiling height** | **in cm** |
| --- | --- |
| Minimum | 160 |
| Maximum | 293 |
| Mean | 218.62 |
| Median | 216 |
| Standard deviation | 26.54 |

| **House height** | **in cm** |
| --- | --- |
| Minimum | 200 |
| Maximum | 390 |
| Mean | 270.77 |
| Median | 270 |
| Standard deviation | 33.4 |

| **Room width** | **in cm** |
| --- | --- |
| Minimum | 110 |
| Maximum | 333 |
| Mean | 222.09 |
| Median | 222 |
| Standard deviation | 48.85 |

| **Room length** | **in cm** |
| --- | --- |
| Minimum | 139 |
| Maximum | 700 |
| Mean | 290.11 |
| Median | 269 |
| Standard deviation | 87.23 |

| **Door width** | **in cm** |
| --- | --- |
| Minimum | 50 |
| Maximum | 86 |
| Mean | 71.07 |
| Median | 72 |
| Standard deviation | 8.91 |

| **Door height** | **in cm** |
| --- | --- |
| Minimum | 160 |
| Maximum | 210 |
| Mean | 175.82 |
| Median | 173 |
| Standard deviation | 11.84 |

| **Window width** | **in cm** |
| --- | --- |
| Minimum | 28 |
| Maximum | 80 |
| Mean | 59.48 |
| Median | 60 |
| Standard deviation | 13.42 |

| **Window height** | **in cm** |
| --- | --- |
| Minimum | 25 |
| Maximum | 90 |
| Mean | 63.32 |
| Median | 62 |
| Standard deviation | 16.27 |

| **Size of the window pectoral** | **in cm** |
| --- | --- |
| Minimum | 60 |
| Maximum | 210 |
| Mean | 113.28 |
| Median | 110 |
| Standard deviation | 27.49 |

| **Size of eave gaps** | **in cm** |
| --- | --- |
| Minimum | 0 |
| Maximum | 20 |
| Mean | 8.12 |
| Median | 10 |
| Standard deviation | 4.81 |

| **Size of gaps above the door** | **in cm** |
| --- | --- |
| Minimum | 0 |
| Maximum | 80 |
| Mean | 4.2 |
| Median | 0 |
| Standard deviation | 13.11 |

| **Size of gaps below the door** | **in cm** |
| --- | --- |
| Minimum | 0 |
| Maximum | 15 |
| Mean | 4.13 |
| Median | 3 |
| Standard deviation | 1.41 |

| **Wall material** | **n** | **Percentage** |
| --- | --- | --- |
| Mud blocks | 49 | 98% |
| Wattle-and-daub | 1 | 2% |

| **Roof structure material** | **n** | **Percentage** |
| --- | --- | --- |
| Wood | 7 | 14% |
| Bamboo | 41 | 82% |
| Poles | 2 | 4% |

| **Roof cover material** | n | Percentage |
| --- | --- | --- |
| Grass | 39 | 78% |
| Metal sheet | 11 | 22% |

| **Interior pavement material** | n | Percentage |
| --- | --- | --- |
| Cement | 9 | 18% |
| Adobe | 23 | 46% |
| None | 18 | 36% |

| **Door panel material** | n | Percentage |
| --- | --- | --- |
| Wood | 48 | 96% |
| Metal | 2 | 4% |

| **Window panel material** | **n** | **Percentage** |
| --- | --- | --- |
| Wood | 14 | 28% |
| Metal | 3 | 6% |
| No window | 6 | 12% |

| **Window screening** | **n** | **Percentage** |
| --- | --- | --- |
| Screened | 4 | 8% |
| Unscreened | 40 | 80% |
| No window | 6 | 12% |

| **Status of the door during survey** | **n** | **Percentage** |
| --- | --- | --- |
| Closed doors | 9 | 18% |
| Opened doors | 41 | 82% |

| **Door screening** | **n** | **Percentage** |
| --- | --- | --- |
| Screened doors | 0 | 0% |
| Unscreened doors | 50 | 100% |

| **Eaves gaps** | **n** | **Percentage** |
| --- | --- | --- |
| Blocked eave gaps | 18 | 36% |
| Open eave gaps | 32 | 64% |
