# Supplementary material for: Innovative house structures for malaria vector control in Nampula district, Mozambique: assessing mosquito entry prevention, indoor comfort, and community acceptance
Source: Front Public Health. 2024 Jun 4;12:1404493. doi: 10.3389/fpubh.2024.1404493 (PMC11183294; doi:10.3389/fpubh.2024.1404493)
Supplement: Supplementary file 2 [file Table_2.docx]

Supplemental Table 2: Floor plans and facades of experimental houses and position of traps and data loggers

| 1. Floor plans and facades of traditional houses |
| --- |
| \|  \| 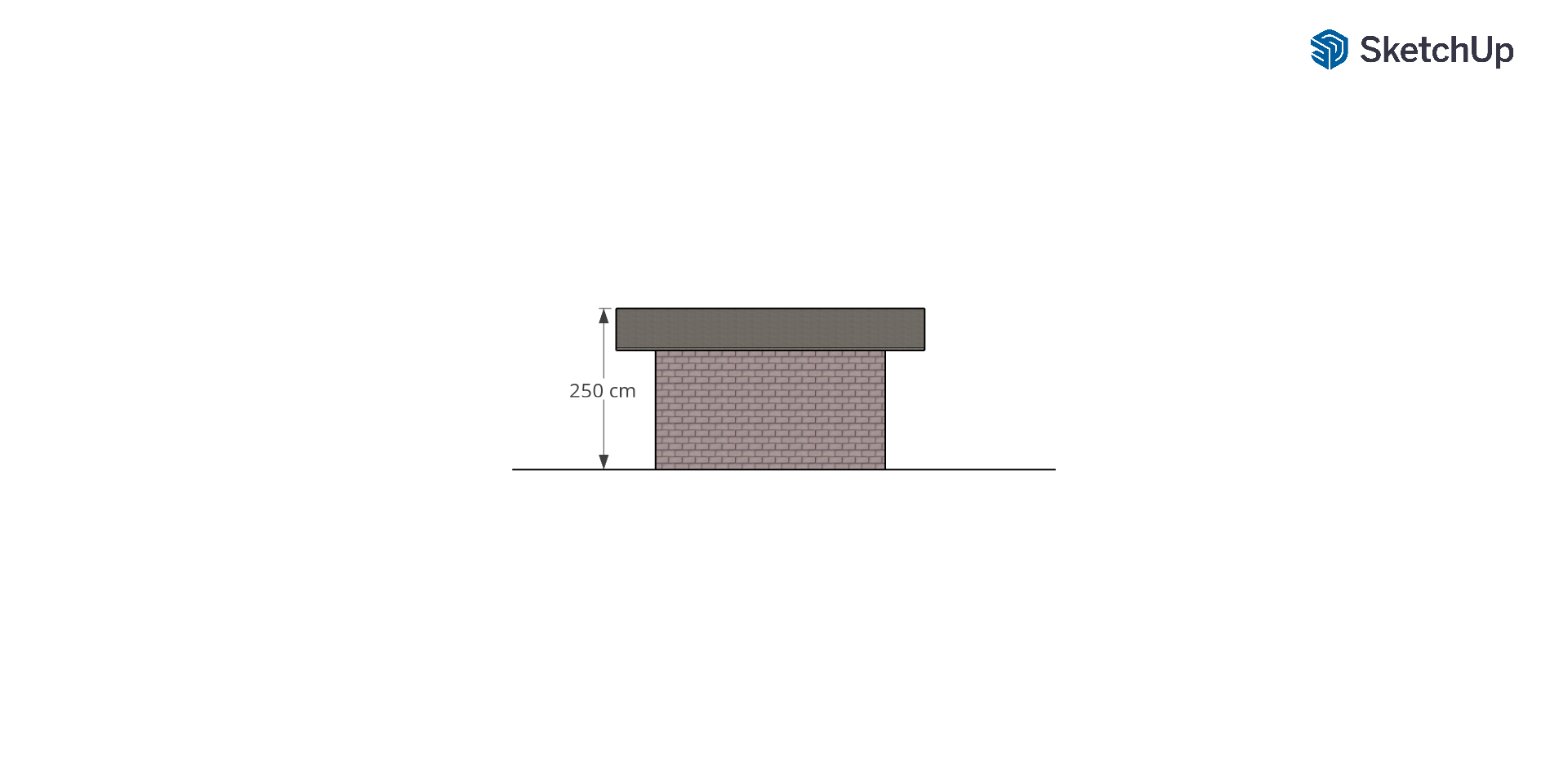 \|  \| \| --- \| --- \| --- \| \|  \| Back facade \|  \| \| 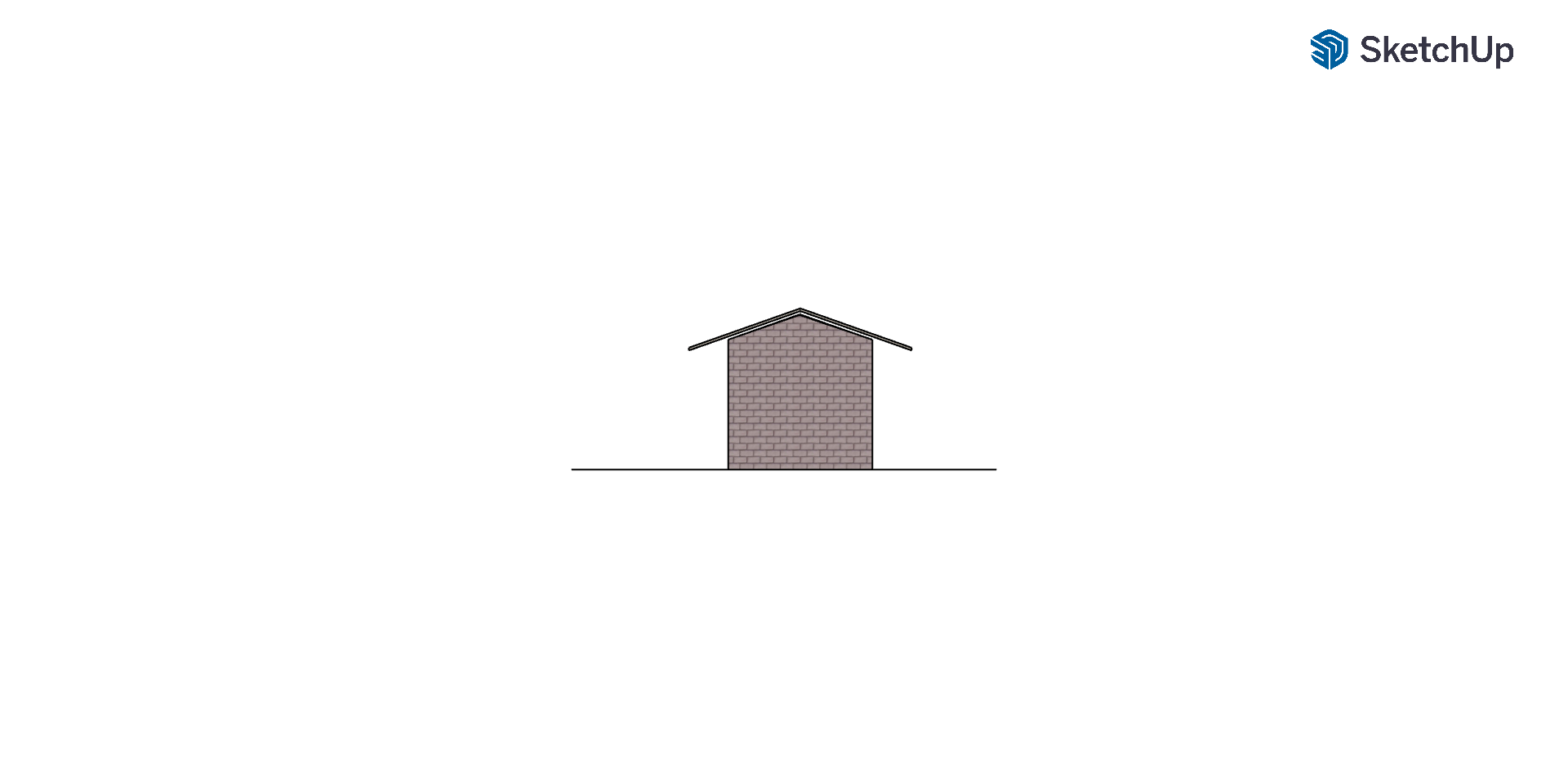 \| 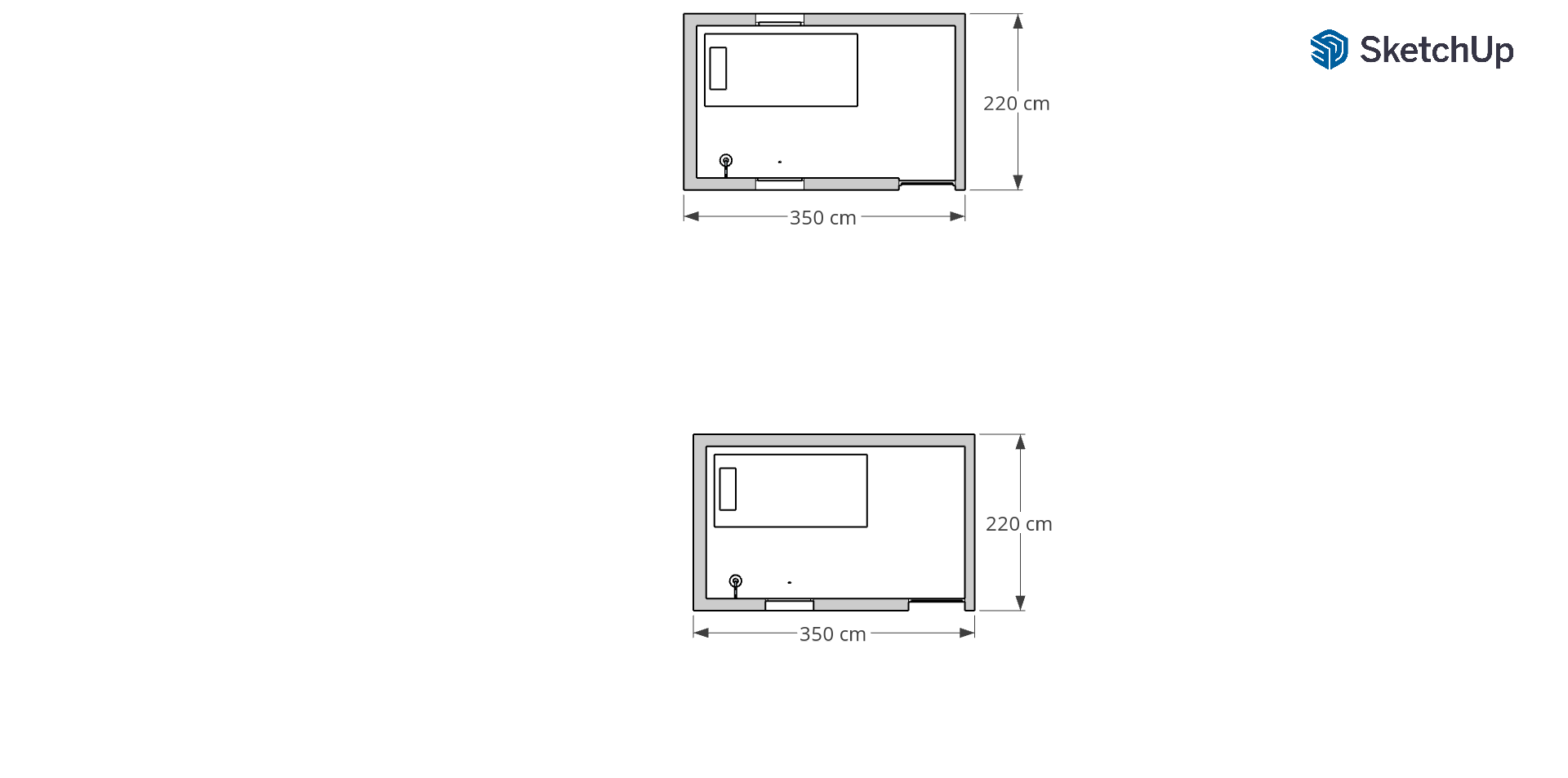 \| 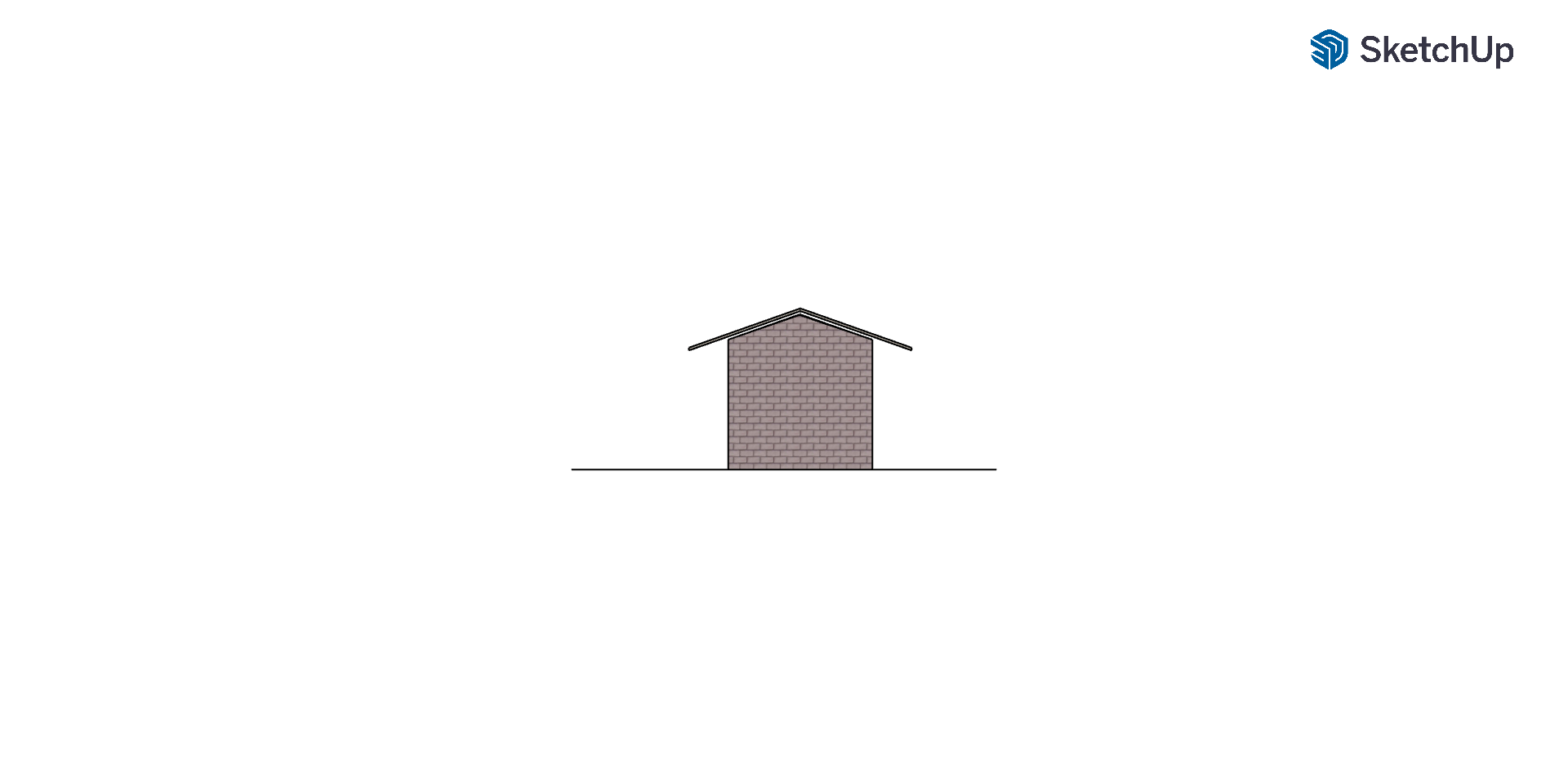 \| \| Left facade \| Floor plan \| Right facade \| \|  \| 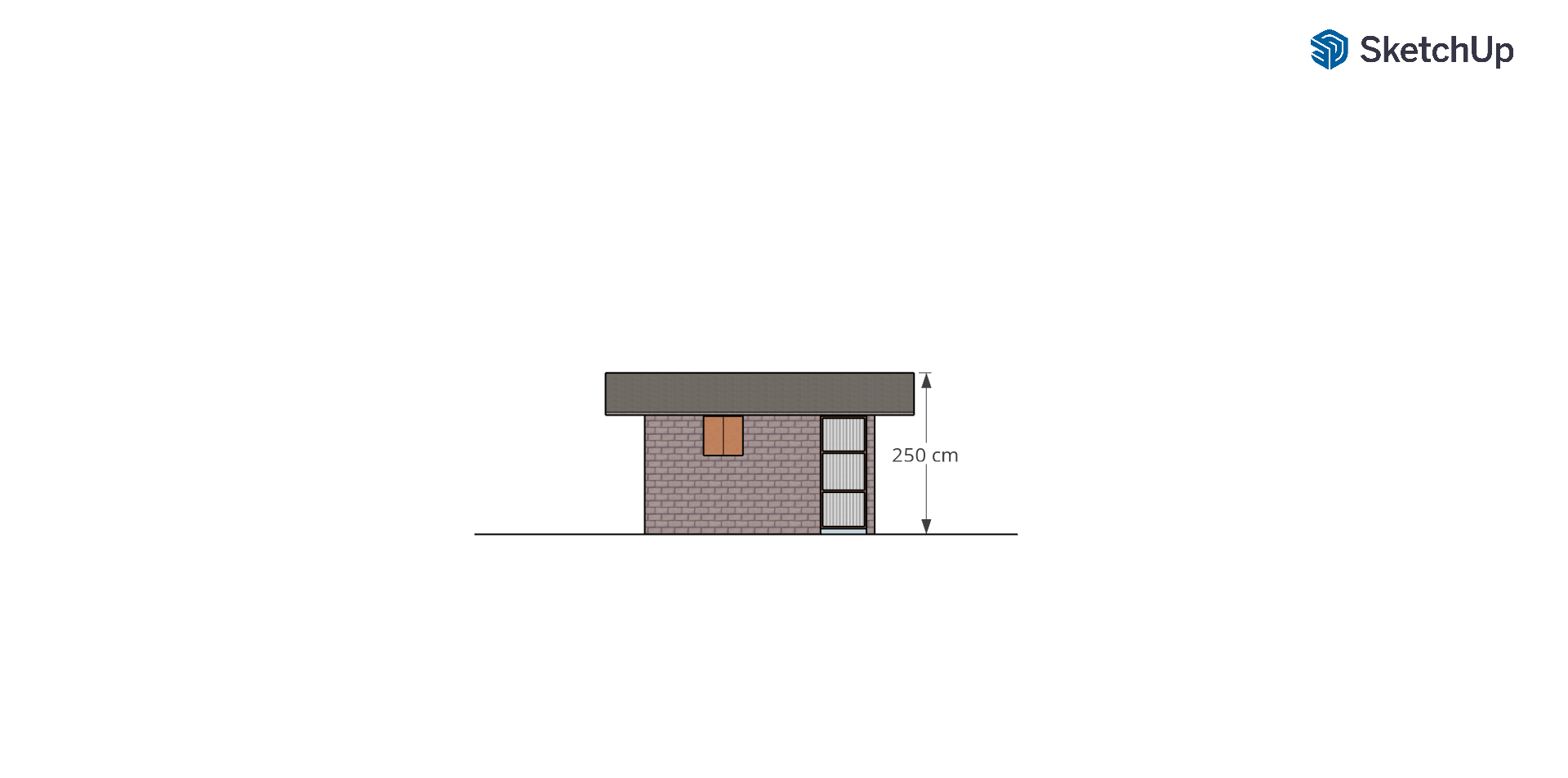 \|  \| \|  \| Front facade \|  \| |
| (B)Floor plans and facades of the modified houses |
| \|  \| 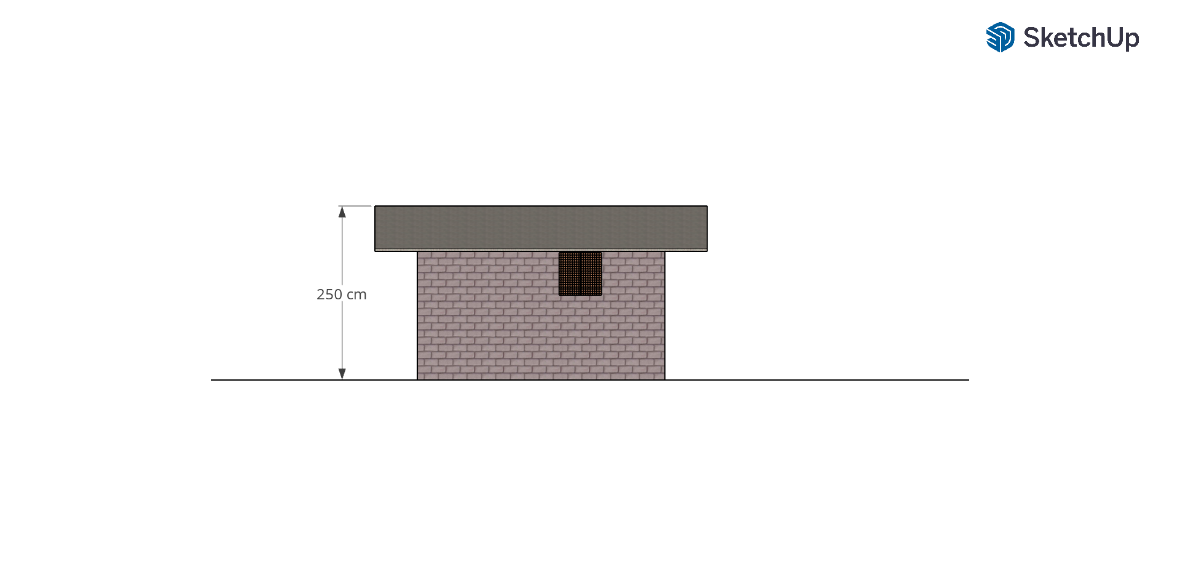 \|  \| \| --- \| --- \| --- \| \|  \| Back facade \|  \| \| 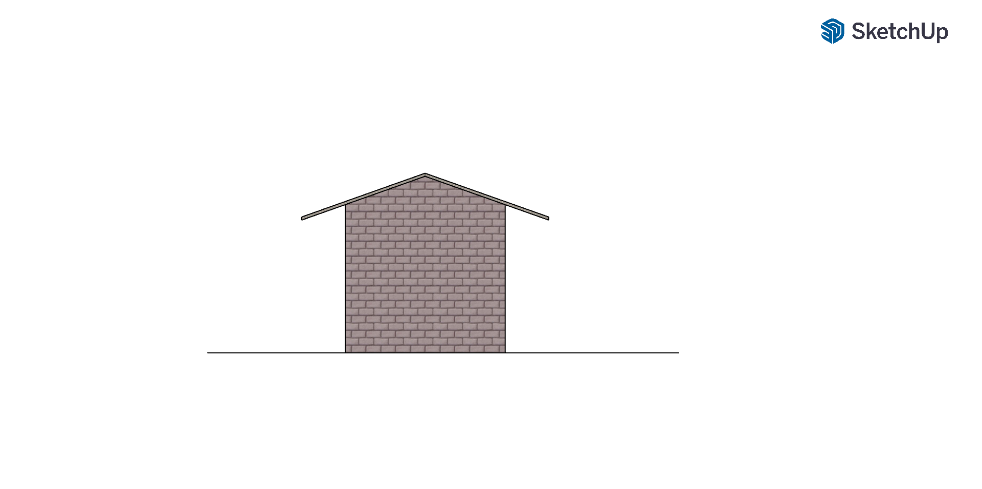 \| 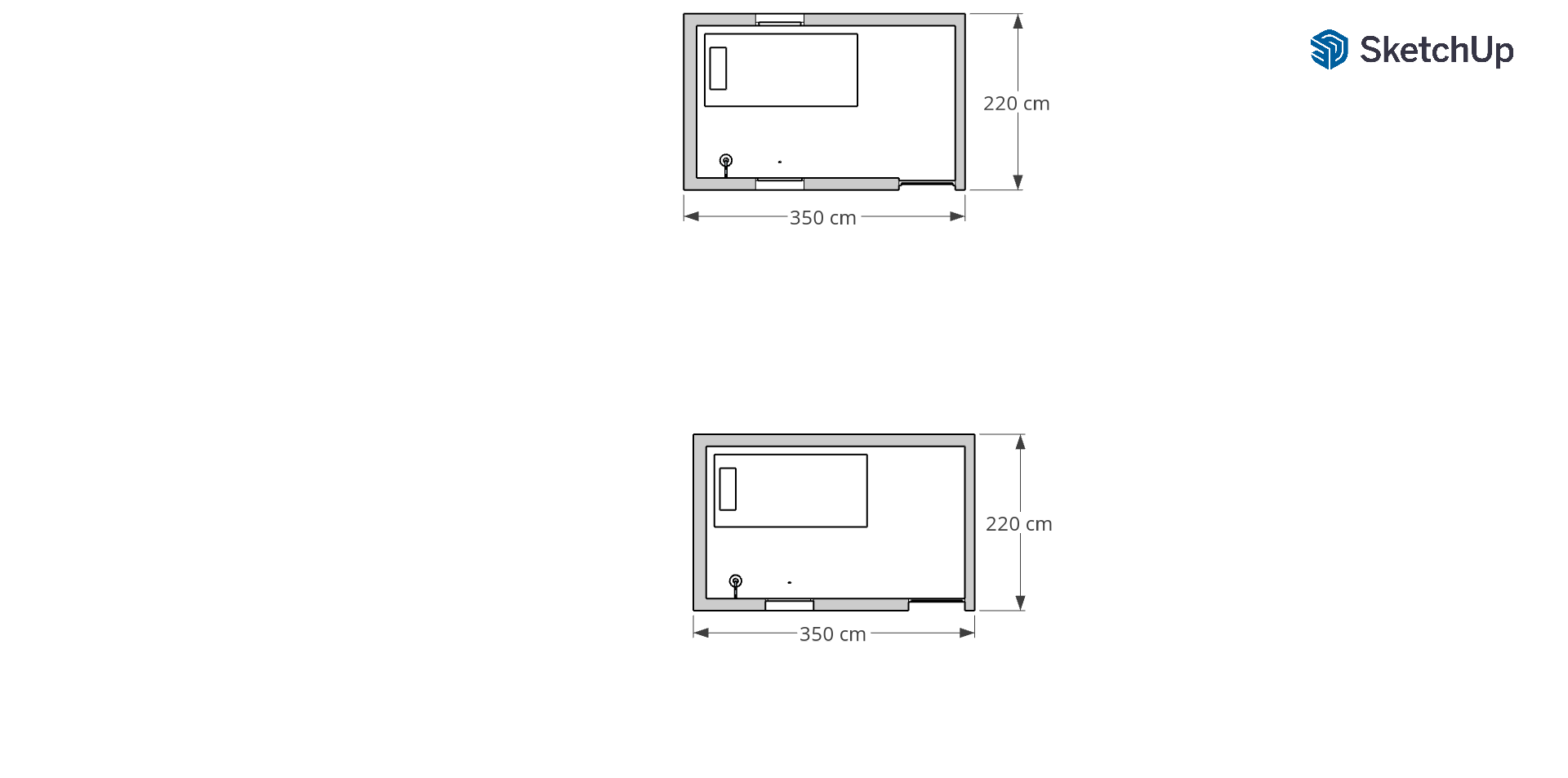 \| 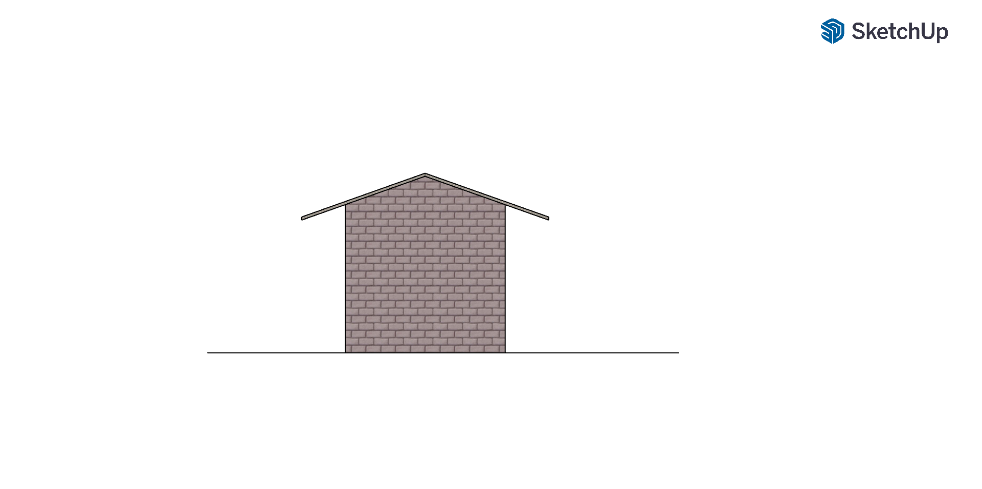 \| \| Left facade \| Floor plan \| Right facade \| \|  \| 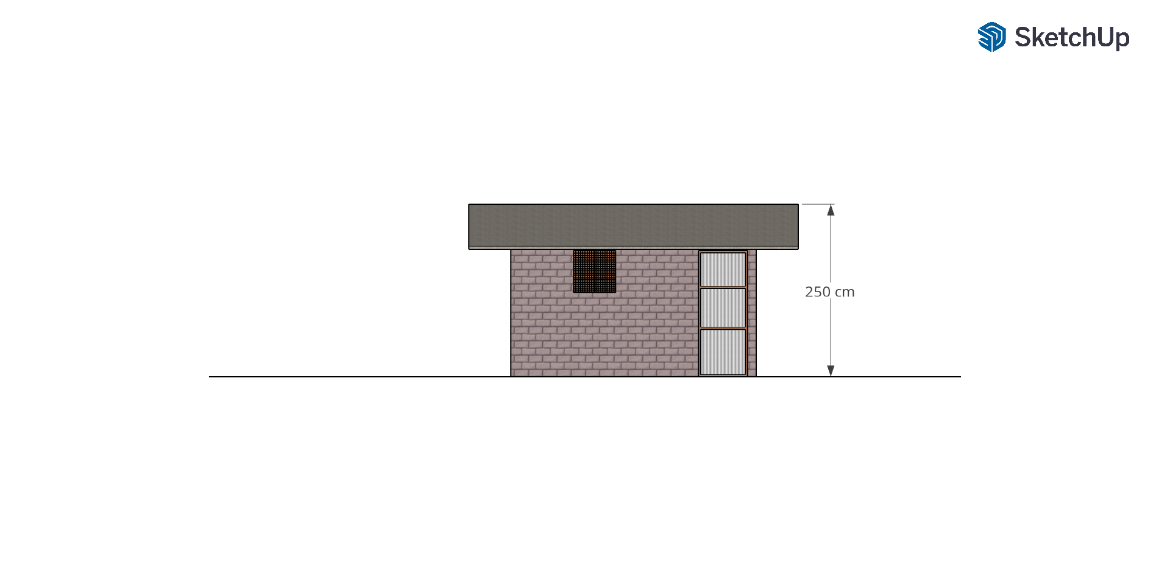 \|  \| \|  \| Front facade \|  \| |

| (C) Position of traps and data loggers in traditional houses |
| --- |
| 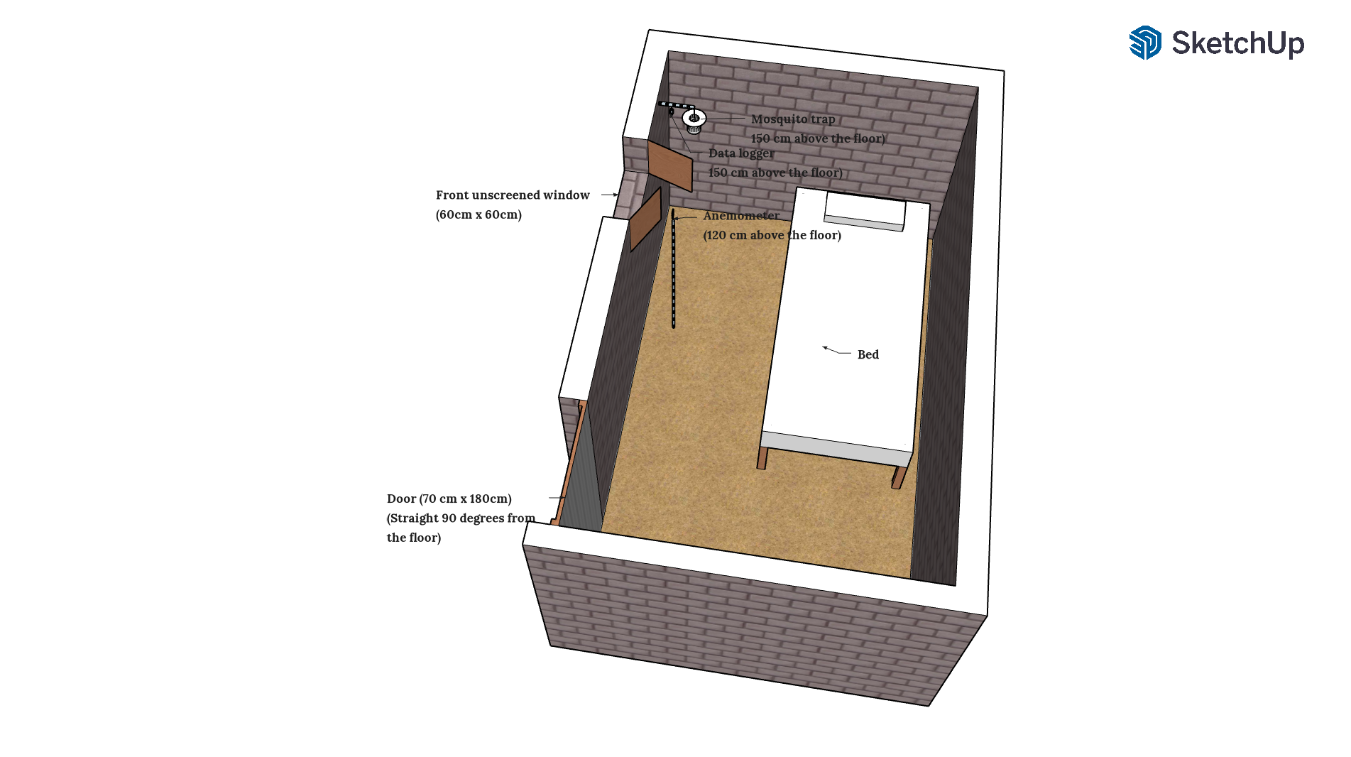 |
| (D) Position of traps and data loggers in modified houses |
| 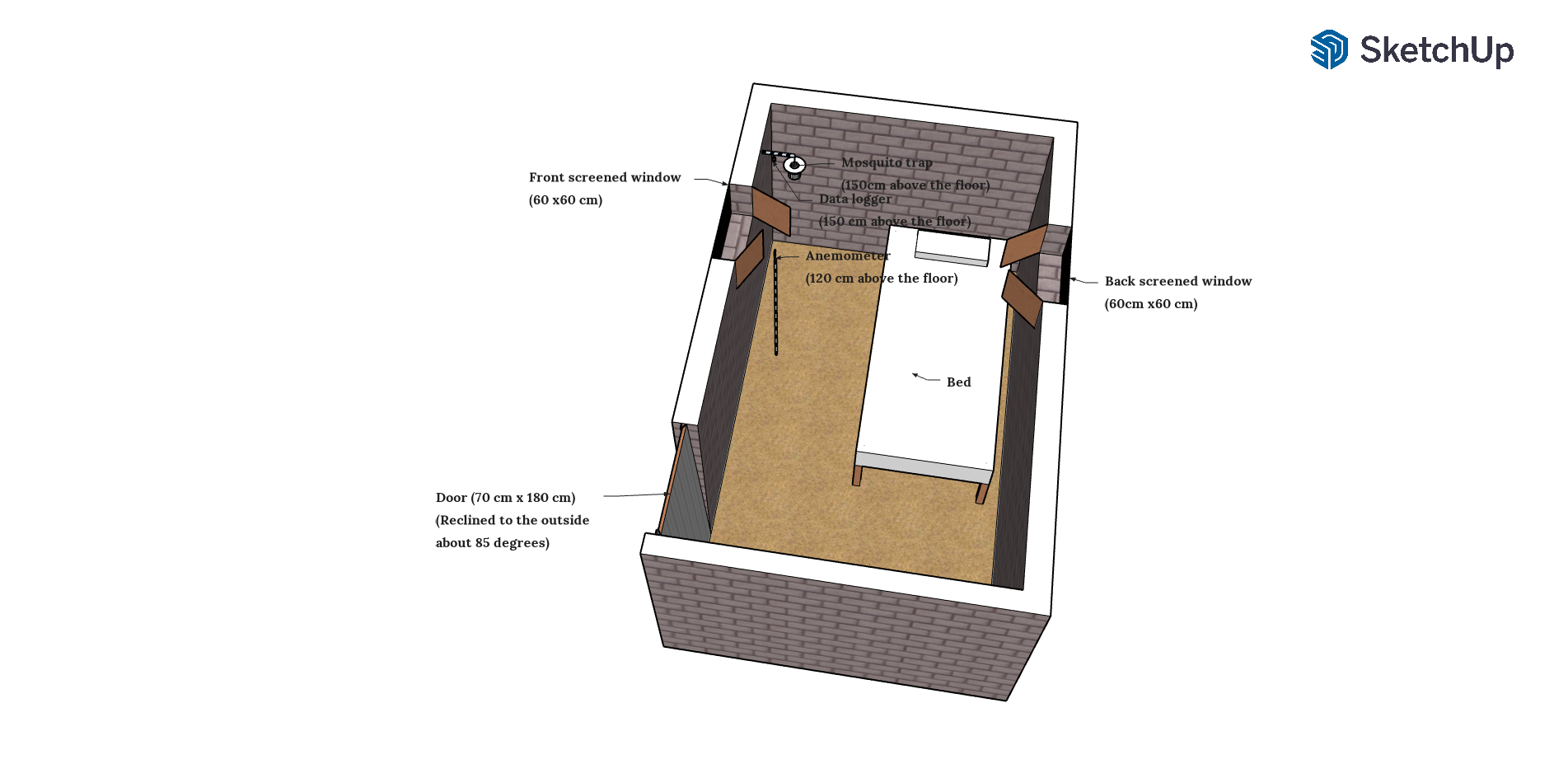 |
