# Supplementary material for: Innovative house structures for malaria vector control in Nampula district, Mozambique: assessing mosquito entry prevention, indoor comfort, and community acceptance
Source: Front Public Health. 2024 Jun 4;12:1404493. doi: 10.3389/fpubh.2024.1404493 (PMC11183294; doi:10.3389/fpubh.2024.1404493)
Supplement: Supplementary file 3 [file Table_3.docx]

Supplemental Table 3: Definition of technical terms used in the study.

**Facade**

A facade refers to any side of a building, also known as a view or a side. Facades can include the front, rear, right, and left sides of a structure.

**Eaves**

Eaves are the areas where a sloping roof meets a wall. They serve to extend over the walls to protect them from rainwater. Walls constructed with mud blocks can be particularly vulnerable to rain damage. Extended eaves significantly increase the lifespan of walls, especially in regions with heavy rainfall.

**Outward Reclining Doors**

Outward reclining doors are installed at a slight angle to the exterior of a house. This innovative approach leverages the principle of gravity to create an automatic closing mechanism for the door. By reclining the door, its center of gravity shifts towards the top, making it inherently unstable. As a result, when the door is opened and released, gravity causes it to swing back and return to its closed position naturally. This automatic closing feature eliminates the need for manual closure or hydraulic springs, ensuring consistent door closure and effectively preventing mosquitoes from entering the house opportunistically. Installing doors reclined outward is simpler if the wall is straight (perpendicular to the ground) or slightly inclined outward. However, installing a door for automated closing can be challenging if the wall is inclined inward.

**Mud Mortar**

Mud mortar is a mixture of clayey soil and water. Builders typically mix it to a point where they consider it optimal for making or laying mud blocks, depending on their needs. In this study, soil extracted from termite mounds was used to prepare the mud mortar.

**Mud Blocks**

Mud blocks, also known as mud bricks, are made from mud mortar using wooden molds. Before molding, the mortar is prepared by saturating soil containing clay with water. In this study, soil extracted from termite mounds was used. Mud blocks are solid and holeless, with dimensions varying based on manufacturing requirements. After molding, mud blocks are dried under the sun for at least a week to eliminate moisture.

**Termite Mounds**

Termite mounds, also known as termitaria, are structures built by termite colonies. These mounds serve as central hubs for termite activity, providing shelter and protection. Constructed primarily from humid soil, saliva, and organic materials, termite mounds regulate internal temperature and humidity levels. Local communities often use termite mound soil to make mud blocks, which offer enhanced wall resistance against cracks and rain compared to soil dug from the ground.

**Slender Bamboo**

Slender bamboo is a type of bamboo found in northern Mozambique. It is thin yet resistant and is commonly available in local markets, sold in bundles.

**Ropes Extracted from Discarded Tires**

Ropes extracted from old tires are repurposed into functional materials for construction projects. In northern Mozambique, tire ropes are used for tying bamboo in roof structures. Extraction involves cutting the inner part of the tire bead with a sharp knife and pulling it through to create ropes.

**Dry Grass**

Dry grass is used as a roofing material by local communities. It is cut fresh from forests or grasslands and left to dry in the sun. Bundles of dried grass are then placed over a plastic mat on the bamboo roof structure, with stems facing upwards and secured with bamboo stakes to prevent displacement. The grass is not treated before use.
