# Supplementary material for: Innovative house structures for malaria vector control in Nampula district, Mozambique: assessing mosquito entry prevention, indoor comfort, and community acceptance
Source: Front Public Health. 2024 Jun 4;12:1404493. doi: 10.3389/fpubh.2024.1404493 (PMC11183294; doi:10.3389/fpubh.2024.1404493)
Supplement: Supplementary file 4 [file Table_4.docx]

Supplemental Table 4: Distribution of mosquito traps. CDC in CDC light trap and NTR is Nitride UV light trap. Empty cell means no trap was allocated in that house at that night.

| Date | T1 | T2 | T3 | T4 | T5 | M1 | M2 | M3 | M4 | M5 |
| --- | --- | --- | --- | --- | --- | --- | --- | --- | --- | --- |
| 2023/01/01 |  |  |  |  |  | CDC | CDC | CDC | CDC | CDC |
| 2023/01/02 | CDC | CDC | CDC | CDC | CDC |  |  |  |  |  |
| 2023/01/03 |  |  |  |  |  | CDC | CDC | CDC | CDC | CDC |
| 2023/01/04 | CDC | CDC | CDC | CDC | CDC |  |  |  |  |  |
| 2023/01/05 |  |  |  |  |  | CDC | CDC | CDC | CDC | CDC |
| 2023/01/06 |  |  |  |  |  | CDC | CDC | CDC | CDC | CDC |
| 2023/01/07 | CDC | CDC | CDC | CDC | CDC |  |  |  |  |  |
| 2023/01/08 | CDC | CDC | CDC | CDC | CDC |  |  |  |  |  |
| 2023/01/09 | CDC | CDC | CDC | CDC | CDC |  |  |  |  |  |
| 2023/01/10 | CDC | CDC | CDC | CDC | CDC | NTR | NTR | NTR | NTR | NTR |
| 2023/01/11 | CDC | CDC | CDC | CDC | CDC | NTR | NTR | NTR | NTR | NTR |
| 2023/01/12 | CDC | CDC | CDC | CDC | CDC | NTR | NTR | NTR | NTR | NTR |
| 2023/01/13 | NTR | NTR | NTR | NTR | NTR | CDC | CDC | CDC | CDC | CDC |
| 2023/01/14 | NTR | NTR | NTR | NTR | NTR | CDC | CDC | CDC | CDC | CDC |
| 2023/01/15 | NTR | NTR | NTR | NTR | NTR | CDC | CDC | CDC | CDC | CDC |
| 2023/01/16 | NTR | NTR | NTR | NTR | NTR | CDC | CDC | CDC | CDC | CDC |
| 2023/01/17 | CDC | CDC | CDC | CDC | CDC | NTR | NTR | NTR | NTR | NTR |
| 2023/01/18 | CDC | CDC | CDC | CDC | CDC | NTR | NTR | NTR | NTR | NTR |
| 2023/01/19 | CDC | CDC | CDC | CDC | CDC | NTR | NTR | NTR | NTR | NTR |
| 2023/01/20 | CDC | CDC | CDC | CDC | CDC | NTR | NTR | NTR | NTR | NTR |
| 2023/01/21 | NTR | NTR | NTR | NTR | NTR | CDC | CDC | CDC | CDC | CDC |
| 2023/01/22 | NTR | NTR | NTR | NTR | NTR | CDC | CDC | CDC | CDC | CDC |
| 2023/01/23 | NTR | NTR | NTR | NTR | NTR | CDC | CDC | CDC | CDC | CDC |
| 2023/01/24 | NTR | NTR | NTR | NTR | NTR | CDC | CDC | CDC | CDC | CDC |
| 2023/01/25 | CDC | CDC | CDC | CDC | CDC | NTR | NTR | NTR | NTR | NTR |
| 2023/01/26 | CDC | CDC | CDC | CDC | CDC | NTR | NTR | NTR | NTR | NTR |
| 2023/01/27 | CDC | CDC | CDC | CDC | CDC | NTR | NTR | NTR | NTR | NTR |
| 2023/01/28 | CDC | CDC | CDC | CDC | CDC | NTR | NTR | NTR | NTR | NTR |
| 2023/01/29 | NTR | NTR | NTR | NTR | NTR | CDC | CDC | CDC | CDC | CDC |
| 2023/01/30 | NTR | NTR | NTR | NTR | NTR | CDC | CDC | CDC | CDC | CDC |
| 2023/01/31 | NTR | NTR | NTR | NTR | NTR | CDC | CDC | CDC | CDC | CDC |
| 2023/02/01 | CDC | CDC | CDC | CDC | CDC | NTR | NTR | NTR | NTR | NTR |
| 2023/02/02 | CDC | CDC | CDC | CDC | CDC | NTR | NTR | NTR | NTR | NTR |
| 2023/02/03 | CDC | CDC | CDC | CDC | CDC | NTR | NTR | NTR | NTR | NTR |
| 2023/02/04 | CDC | CDC | CDC | CDC | CDC | NTR | NTR | NTR | NTR | NTR |
| 2023/02/05 | CDC | CDC | CDC | CDC | CDC | NTR | NTR | NTR | NTR | NTR |
| 2023/02/06 | CDC | CDC | CDC | CDC | CDC | NTR | NTR | NTR | NTR | NTR |
| 2023/02/07 | CDC | CDC | CDC | CDC | CDC | NTR | NTR | NTR | NTR | NTR |
| 2023/02/08 | NTR | NTR | NTR | NTR | NTR | CDC | CDC | CDC | CDC | CDC |
| 2023/02/09 | NTR | NTR | NTR | NTR | NTR | CDC | CDC | CDC | CDC | CDC |
| 2023/02/10 | NTR | NTR | NTR | NTR | NTR | CDC | CDC | CDC | CDC | CDC |
| 2023/02/11 | NTR | NTR | NTR | NTR | NTR | CDC | CDC | CDC | CDC | CDC |
| 2023/02/12 | CDC | CDC | CDC | CDC | CDC | NTR | NTR | NTR | NTR | NTR |
| 2023/02/13 | CDC | CDC | CDC | CDC | CDC | NTR | NTR | NTR | NTR | NTR |
| 2023/02/14 | CDC | CDC | CDC | CDC | CDC | NTR | NTR | NTR | NTR | NTR |
| 2023/02/15 | CDC | CDC | CDC | CDC | CDC | NTR | NTR | NTR | NTR | NTR |
| 2023/02/16 | NTR | NTR | NTR | NTR | NTR | CDC | CDC | CDC | CDC | CDC |
| 2023/02/17 | NTR | NTR | NTR | NTR | NTR | CDC | CDC | CDC | CDC | CDC |
| 2023/02/18 | NTR | NTR | NTR | NTR | NTR | CDC | CDC | CDC | CDC | CDC |
| 2023/02/19 | NTR | NTR | NTR | NTR | NTR | CDC | CDC | CDC | CDC | CDC |
| 2023/02/20 | NTR | NTR | NTR | NTR | NTR | CDC | CDC | CDC | CDC | CDC |
| 2023/02/21 | CDC | CDC | CDC | CDC | CDC | NTR | NTR | NTR | NTR | NTR |
| 2023/02/22 | CDC | CDC | CDC | CDC | CDC | NTR | NTR | NTR | NTR | NTR |
| 2023/02/23 | CDC | CDC | CDC | CDC | CDC | NTR | NTR | NTR | NTR | NTR |
| 2023/02/24 | CDC | CDC | CDC | CDC | CDC | NTR | NTR | NTR | NTR | NTR |
| 2023/02/25 | CDC | CDC | CDC | CDC | CDC | NTR | NTR | NTR | NTR | NTR |
| 2023/02/26 | NTR | NTR | NTR | NTR | NTR | CDC | CDC | CDC | CDC | CDC |
| 2023/02/27 | NTR | NTR | NTR | NTR | NTR | CDC | CDC | CDC | CDC | CDC |
| 2023/02/28 | NTR | NTR | NTR | NTR | NTR | CDC | CDC | CDC | CDC | CDC |
| 2023/03/01 | NTR | NTR | NTR | NTR | NTR | CDC | CDC | CDC | CDC | CDC |
| 2023/03/02 | NTR | NTR | NTR | NTR | NTR | CDC | CDC | CDC | CDC | CDC |
| 2023/03/03 | CDC | CDC | CDC | CDC | CDC | NTR | NTR | NTR | NTR | NTR |
| 2023/03/04 | CDC | CDC | CDC | CDC | CDC | NTR | NTR | NTR | NTR | NTR |
| 2023/03/05 | CDC | CDC | CDC | CDC | CDC | NTR | NTR | NTR | NTR | NTR |
| 2023/03/06 | CDC | CDC | CDC | CDC | CDC | NTR | NTR | NTR | NTR | NTR |
| 2023/03/07 | CDC | CDC | CDC | CDC | CDC | NTR | NTR | NTR | NTR | NTR |
| 2023/03/08 | NTR | NTR | NTR | NTR | NTR | CDC | CDC | CDC | CDC | CDC |
| 2023/03/09 | NTR | NTR | NTR | NTR | NTR | CDC | CDC | CDC | CDC | CDC |
| 2023/03/10 | NTR | NTR | NTR | NTR | NTR | CDC | CDC | CDC | CDC | CDC |
| 2023/03/11 | NTR | NTR | NTR | NTR | NTR | CDC | CDC | CDC | CDC | CDC |
| 2023/03/12 | NTR | NTR | NTR | NTR | NTR | CDC | CDC | CDC | CDC | CDC |
| 2023/03/13 | CDC | CDC | CDC | CDC | CDC | NTR | NTR | NTR | NTR | NTR |
| 2023/03/14 | CDC | CDC | CDC | CDC | CDC | NTR | NTR | NTR | NTR | NTR |
| 2023/03/15 | CDC | CDC | CDC | CDC | CDC | NTR | NTR | NTR | NTR | NTR |
| 2023/03/16 | CDC | CDC | CDC | CDC | CDC | NTR | NTR | NTR | NTR | NTR |
| 2023/03/17 | CDC | CDC | CDC | CDC | CDC | NTR | NTR | NTR | NTR | NTR |
| 2023/03/18 | NTR | NTR | NTR | NTR | NTR | CDC | CDC | CDC | CDC | CDC |
| 2023/03/19 | NTR | NTR | NTR | NTR | NTR | CDC | CDC | CDC | CDC | CDC |
| 2023/03/20 | NTR | NTR | NTR | NTR | NTR | CDC | CDC | CDC | CDC | CDC |
| 2023/03/21 | NTR | NTR | NTR | NTR | NTR | CDC | CDC | CDC | CDC | CDC |
| 2023/03/22 | NTR | NTR | NTR | NTR | NTR | CDC | CDC | CDC | CDC | CDC |
| 2023/03/23 | CDC | CDC | CDC | CDC | CDC | NTR | NTR | NTR | NTR | NTR |
| 2023/03/24 | CDC | CDC | CDC | CDC | CDC | NTR | NTR | NTR | NTR | NTR |
| 2023/03/25 | CDC | CDC | CDC | CDC | CDC | NTR | NTR | NTR | NTR | NTR |
| 2023/03/26 | CDC | CDC | CDC | CDC | CDC | NTR | NTR | NTR | NTR | NTR |
| 2023/03/27 | CDC | CDC | CDC | CDC | CDC | NTR | NTR | NTR | NTR | NTR |
| 2023/03/28 | NTR | NTR | NTR | NTR | NTR | CDC | CDC | CDC | CDC | CDC |
| 2023/03/29 | NTR | NTR | NTR | NTR | NTR | CDC | CDC | CDC | CDC | CDC |
| 2023/03/30 | NTR | NTR | NTR | NTR | NTR | CDC | CDC | CDC | CDC | CDC |
| 2023/03/31 | NTR | NTR | NTR | NTR | NTR | CDC | CDC | CDC | CDC | CDC |
