# Supplementary material for: Innovative house structures for malaria vector control in Nampula district, Mozambique: assessing mosquito entry prevention, indoor comfort, and community acceptance
Source: Front Public Health. 2024 Jun 4;12:1404493. doi: 10.3389/fpubh.2024.1404493 (PMC11183294; doi:10.3389/fpubh.2024.1404493)
Supplement: Supplementary file 5 [file Table_5.docx]

Supplemental Table 5. Statistical test from analysis of variance of environmental parameters between each group of traditional or modified houses. Values in the table are p-values.

| House | Temperature | Relative humidity | Carbon dioxide | Wind | Anopheles | Non-Anopheles |
| --- | --- | --- | --- | --- | --- | --- |
| Traditional | 0.634 | 0.694 | 0.395 | 0.026 | 0.390 | 0.958 |
| Modified | 0.888 | 0.869 | 0.787 | <0.000 | 0.489 | 0.497 |
