# Supplementary material for: Innovative house structures for malaria vector control in Nampula district, Mozambique: assessing mosquito entry prevention, indoor comfort, and community acceptance
Source: Front Public Health. 2024 Jun 4;12:1404493. doi: 10.3389/fpubh.2024.1404493 (PMC11183294; doi:10.3389/fpubh.2024.1404493)
Supplement: Supplementary file 6 [file Table_6.docx]

Supplemental Table 6: Assessment of construction workers’ experience on constructing modified houses.

| Number | Question | Response | Percentage of respondents |
| --- | --- | --- | --- |
| 1 | For how many years you have been working with constructions? | < 1 year | 0 |
|  |  | 1 to 5 years | 40 |
|  |  | 5 to 10 years | 20 |
|  |  | > 10 years | 40 |
| 2 | Do you have formal technical instruction or education in construction? | Yes | 10 |
|  |  | No | 90 |
| 3 | What kind of constructions you are more experienced with? | Adobe based | 30 |
|  |  | Wattle and daub based | 0 |
|  |  | Cement base | 0 |
|  |  | All | 70 |
| 4 | Have you ever built a house with purposes of blocking mosquito entry? | Yes | 40 |
|  |  | No | 60 |
| 5 | How was it for you to set the house orientation so that the Windows face the direction for the highest wind speed? | Very easy | 10 |
|  |  | Easy | 80 |
|  |  | Difficult | 10 |
|  |  | Very difficult | 0 |
| 6 | How was it for you to add mosquito net on the windows? | Very easy | 60 |
|  |  | Easy | 40 |
|  |  | Difficult | 0 |
|  |  | Very difficult | 0 |
| 7 | How was it for you to recline the door to the front? | Very easy | 10 |
|  |  | Easy | 40 |
|  |  | Difficult | 30 |
|  |  | Very difficult | 20 |
| 8 | How was it for you to put a door opening stopper? | Very easy | 10 |
|  |  | Easy | 60 |
|  |  | Difficult | 10 |
|  |  | Very difficult | 10 |
| 9 | How was it for you to close the eaves with mud? | Very easy | 0 |
|  |  | Easy | 50 |
|  |  | Difficult | 20 |
|  |  | Very difficult | 30 |
| 10 | Which modifications you think to be able to implemente alone withouth further supervision? | Screened windows | 0 |
|  |  | Closing eaves with mud | 0 |
|  |  | Reclining the door | 0 |
|  |  | Door opening stopper | 0 |
|  |  | House orientation | 0 |
|  |  | All | 100 |
|  |  | None | 0 |
| 11 | Which modifications you found more difficult to implement in you experience here at the mosquito village? | Screened windows | 0 |
|  |  | Closing eaves with mud | 60 |
|  |  | Reclining the door | 20 |
|  |  | Door opening stopper | 0 |
|  |  | House orientation | 0 |
|  |  | All | 0 |
|  |  | None | 20 |
