# Supplementary figures and images for: Innovative house structures for malaria vector control in Nampula district, Mozambique: assessing mosquito entry prevention, indoor comfort, and community acceptance
Source: Front Public Health. 2024 Jun 4;12:1404493. doi: 10.3389/fpubh.2024.1404493 (PMC11183294; doi:10.3389/fpubh.2024.1404493)

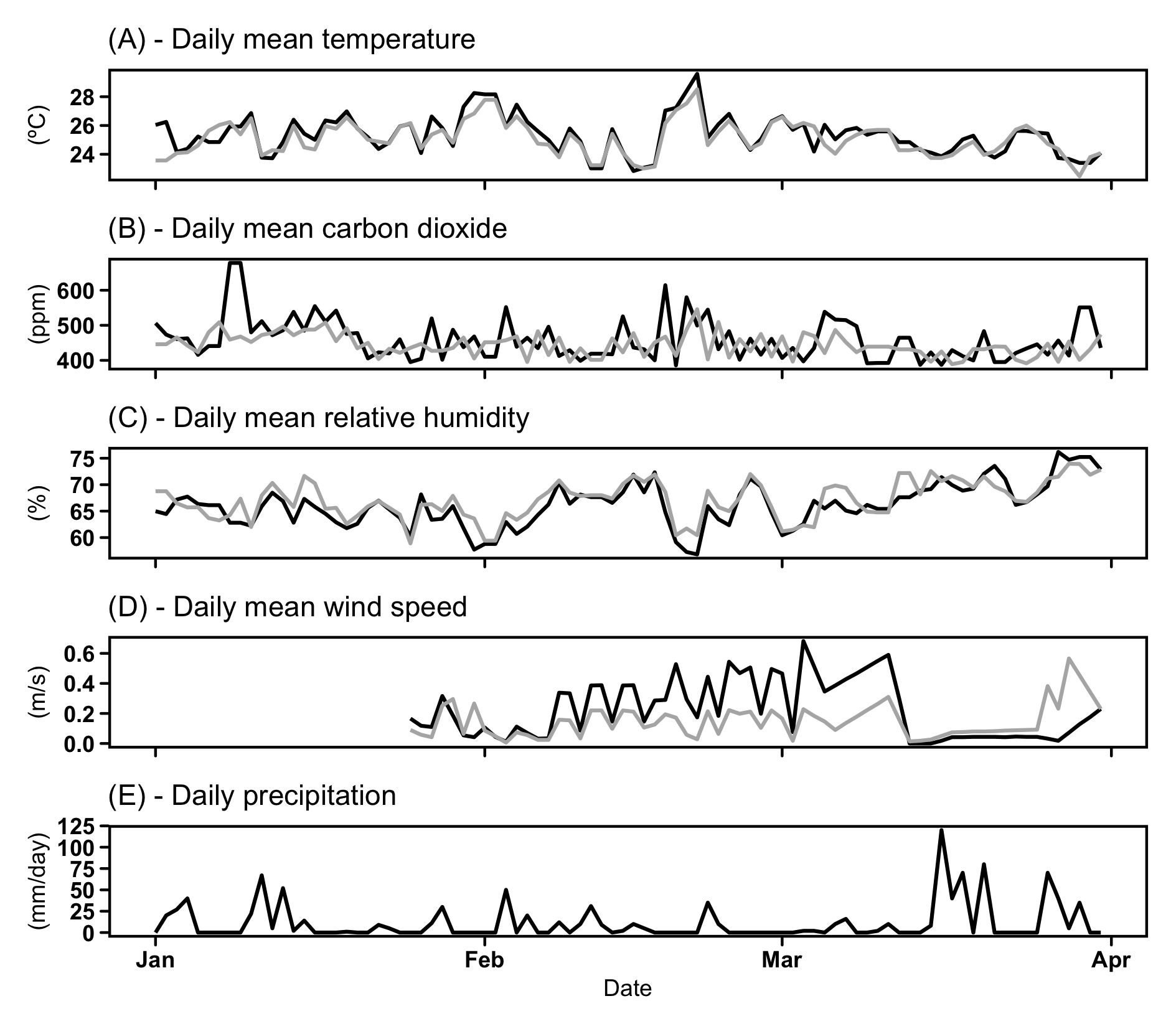

Supplement: SUPPLEMENTARY IMAGE 1 — Daily mean values of environmental factors. Gray solid line is traditional house and black solid line is modified house. (A) is daily mean temperature in degrees Celsius. (B) is daily mean carbon dioxide in parts per million. (C) is daily mean relative humidity in percentage. (D) is daily mean wind speed in meters per second. (E) is daily precipitation in millimeters per day. All environmental factors were measured indoors except for precipitation measured outdoors. [file Image_1.tif]
